# DIRECT: RNA contact predictions by integrating structural patterns

**DOI:** 10.1186/s12859-019-3099-4

**Published:** 2019-10-15

**Authors:** Yiren Jian, Xiaonan Wang, Jaidi Qiu, Huiwen Wang, Zhichao Liu, Yunjie Zhao, Chen Zeng

**Affiliations:** 1 Institute of Biophysics and Department of Physics, Central China Normal University, Wuhan, 430079 China; 2 Department of Physics, The George Washington University, Washington DC, 20052 USA

**Keywords:** RNA, Restricted Boltzmann machine, Coevolution, Nucleotide-nucleotide interaction

## Abstract

**Background:**

It is widely believed that tertiary nucleotide-nucleotide interactions are essential in determining RNA structure and function. Currently, direct coupling analysis (DCA) infers nucleotide contacts in a sequence from its homologous sequence alignment across different species. DCA and similar approaches that use sequence information alone typically yield a low accuracy, especially when the available homologous sequences are limited. Therefore, new methods for RNA structural contact inference are desirable because even a single correctly predicted tertiary contact can potentially make the difference between a correct and incorrectly predicted structure. Here we present a new method DIRECT (Direct Information REweighted by Contact Templates) that incorporates a Restricted Boltzmann Machine (RBM) to augment the information on sequence co-variations with structural features in contact inference.

**Results:**

Benchmark tests demonstrate that DIRECT achieves better overall performance than DCA approaches. Compared to mfDCA and plmDCA, DIRECT produces a substantial increase of 41 and 18%, respectively, in accuracy on average for contact prediction. DIRECT improves predictions for long-range contacts and captures more tertiary structural features.

**Conclusions:**

We developed a hybrid approach that incorporates a Restricted Boltzmann Machine (RBM) to augment the information on sequence co-variations with structural templates in contact inference. Our results demonstrate that DIRECT is able to improve the RNA contact prediction.

## Background

RNA molecules play critical roles in various biological processes [1–8]. Therefore, a comprehensive determination of RNA structure is critical to understanding structure-function relationships. Unfortunately, it is still challenging to precisely determine structure from direct experimentation [9]. In response, many computational RNA tertiary structure prediction methods have been developed, including homology or fragments-based prediction (ModeRNA, Vfold, RNAComposer, 3dRNA) [10–16] and simulation-based prediction (SimRNA, Rosetta FARFAR, iFoldRNA, NAST) [17–21]. Using these strategies, sequence and secondary structure information can be used to predict RNA tertiary structures. The secondary structure is able to define the stem regions and single-stranded loops but leaves RNA tertiary topology unaddressed. Although prediction accuracy has been improved over the years, the tertiary prediction task remains challenging for large RNAs with complex topology. One promising approach is to first predict the tertiary contacts (loop-loop contacts and contacts in junction regions) and then use these interactions to predict the RNA structure. The starting point for this approach is to determine the potential contacts themselves.

One can exploit what is known about nucleotide-nucleotide interactions from experimental studies to heuristically provide data about the distances involved in such interactions. One of the most successful methods for contact prediction, based on this approach, is direct coupling analysis (DCA). DCA infers the interacting nucleotides in a sequence from the sequence coevolution across different species [22–33]. A recent mean-field formulation of DCA (mfDCA) provides an efficient computational framework to extract direct contact information and has been applied to many RNAs. It has been shown that DCA provides sufficient native intra-domain and inter-domain nucleotide-nucleotide contact information for riboswitch and RNA-protein complexes [34–36]. Another inference method called plmDCA, which maximizes the pseudo-likelihood instead of using the mean-field approximation for maximizing the likelihood, improves the contact predictions [37]. In addition to DCA, there are also network-based or machine learning approaches to infer covariation signals from multiple sequence alignments [38–45]. The feature common to these approaches is the exclusive use of evolutionary information extracted from homologous sequences. The prediction accuracy thus depends on accurate multiple sequence alignments of a thousand or more homologous sequences.

An alternative to contact prediction from sequence co-variations is to incorporate structural information as well. Skwark et al. applied a pattern-recognition approach to the contact prediction of a residue pair by examining the expected pattern of nearby contacts surrounding the pair [46]. Specifically, a 3 × 3 matrix of local contacts is constructed as follows. Each residue of the pair is expanded into a fragment of three residues by including the two neighbors, and all residue-residue contacts between the two fragments form the 3 × 3 matrix with element value of 1 for contact and 0 for non-contact. It was found that a contact at the center of the 3 × 3 matrix is typically surrounded by three other contacts in the matrix and a non-contact at the center. However, a contact at the center is likely surrounded by no more than one other contact. By incorporating these local contact patterns, this pattern-recognition approach is able to improve the prediction of alpha helices and beta strands for protein secondary structures.

However, it is more important and difficult to pinpoint the RNA interactions in loop-loop and junction regions than to identify its secondary structure of base-pair interactions. Existing methods on proteins only consider local structural patterns modeled as statistical potential. This approach ignores global structural features that might be useful in improving the RNA prediction accuracy. Therefore, we introduce a new method that first learns a lookup table of contact weights by a Restricted Boltzmann Machine (RBM) from non-redundant and known RNA structures. Then, this lookup table is used to improve RNA contact prediction obtained from sequence co-evolution by DCA. We call our method Direct Information REweighted by Contact Templates (DIRECT). In a benchmark testing on riboswitch, DIRECT outperforms the state-of-the-art DCA predictions for long-range contacts and loop-loop contacts. Moreover, DIRECT maintains better predictions when the number of available sequences is limited. Here, we examined the accuracy of contact prediction for the 5 RNAs using only 50 randomly chosen homologous sequences that represent about 11 to 43% of all available sequences for the 5 RNAs.

## Results

### DIRECT achieves better overall performance

Traditional direct coupling analysis (DCA) for RNA contact prediction has some drawbacks. For one, DCA requires a sufficient number of homologous sequences for accurate sequence co-evolution analysis, which may not be readily available. Moreover, a co-evolving pair of nucleotides can interact within the same molecule (intra-molecule) or across the homodimer interface (inter-molecule) of the RNA. In addition, several unknown factors, other than intra- or inter-molecular interactions, can result in co-evolving pairs and make it difficult to detect the true contacts among the evolving pairs without additional information. One way to overcome this difficulty is to augment the contact detection of a target RNA sequence with additional information on the structural contact template expected of the RNA class to which the target RNA belongs. To this end, we employ a Restricted Boltzmann Machine to learn the contact template of RNAs by using the structures and then improve the contact predictions (Additional file 1: Figure S1).

**Fig. 1:**
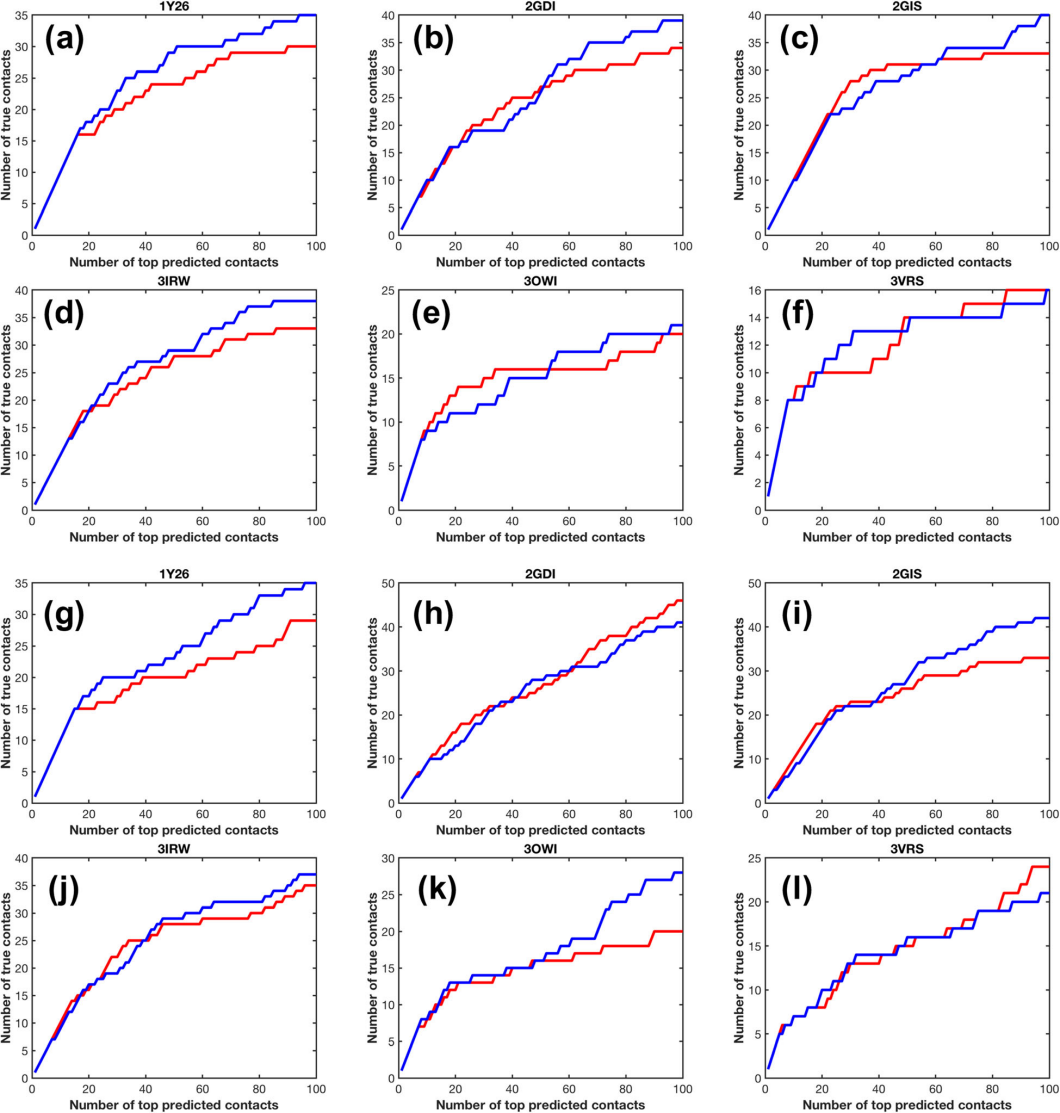
DIRECT vs. DCA. Accuracy of nucleotide-nucleotide contact prediction for all six RNAs in the testing set. **a**, **b**, **c**, **d**, **e** and **f** Comparison between DIRECT and mfDCA. The number of true contacts among the top predicted contacts is shown for each of the six RNAs. Except for 3VRS, DIRECT (blue lines) achieves 13% higher true positive on average than mfDCA (red lines) for true contacts among the top 100 predicted contacts. **g**, **h**, **I**, **j**, **k** and **l** Comparison between DIRECT and plmDCA. DIRECT (blue lines) achieves 11% higher true positive on average than plmDCA (red lines) for true contacts among the top 100 predicted contacts

We used a published riboswitch benchmark dataset to evaluate DIRECT described in Methods (Additional file 1: Table S1, Figure S2) [34]. Six target RNAs are tested as shown in Fig. 1a, b, c, d, e, f. For a given target RNA, the RNA itself and its homologs are removed from the training set. We compare the success rate of mfDCA and DIRECT in predicting the true intra-molecular contacts from the top detected co-evolving pairs (up to top 100). As shown in Fig. 1a, b, c, d, e, f, DIRECT is 5%~ 7% more precise (positive predictive value defined in Methods) than mfDCA for 1Y26, 2GDI, 2GIS, and 3IRW predictions. There is also a slight increase by 2% for 3OWI prediction. The improvement continued beyond the top 100 pairs. The only exception is 3VRS, for it differs from others by its higher-order RNA architecture stabilized by pseudoknots with few standard Watson-Crick pairs, which may lead to a low accuracy for contact prediction. The average increase in true positive is 13%. We further evaluated our method DIRECT comparing it to plmDCA, an algorithm that infers the direct coupling using pseudo-likelihood maximization. As shown in Fig. 1g, h, i, j, k, l, DIRECT is 6%~ 8% more precise (positive predictive value defined in Methods) than plmDCA for 1Y26, 2GIS, and 3OWI predictions. There is also a slight increase by 2% for 3IRW prediction. Though DIRECT produces lower PPV in 2GDI and 3VRS, DIRECT has 11% more true positive on average.

### DIRECT improves predictions for long-range contacts

A contact range measures the sequence distance between the two nucleotides in the contact. Contacts at different ranges convey different information. Short-range contacts in an RNA molecule reflect its local secondary structure. Long-range contacts are base pairs whose contact is based on folding back, loop-loop, or junction interactions. The loop-loop and junction interactions dictate the RNA topology of its structure and are likely to be more useful than secondary structure pairs in structure prediction. A slight improvement in long-range contact prediction, therefore, can have a significant impact on the accuracy and speed of RNA tertiary structure modeling because long-range contacts drastically reduce the structural space that needs to be searched for modeling. Prediction based on long-range contacts remains difficult for most traditional methods. DCA predicts more accurately for short- (5~12 nt) and medium-range (13~24 nt) contacts, but less accurately for long-range (24 nt+) contacts. DIRECT, however, utilizes the structural contact template to re-rank DCA predictions and is able to improve the long-range contact prediction (shown in Additional file 1: Table S2).

### DIRECT captures more tertiary structural features

The interaction types between different RNA secondary structure elements vary significantly. According to Chargaff’s second parity rule, base-pair contacts are easier to predict. It remains difficult to predict long-range tertiary contacts. DIRECT is designed to capture the structural contact and improve the prediction accuracy for long-range tertiary contacts. To verify this, we divided the tertiary contacts into four categories: stem-loop, loop-loop, intra stem-stem, and inter stem-stem contacts. The intra stem-stem contacts between two nucleotides in the same stem determine the stem topology such as bending or twisting. On the other hand, contacts of stem-loop, loop-loop, and inter stem-stem can be used as distance constraints on the RNA tertiary fold.

In Additional file 1: Table S3, it can be seen that the largest improvement of predictions by DIRECT lies in tertiary structural contacts. The correct prediction of base pairs can determine RNA secondary structure. The prediction accuracies of base pairs are similar between DCA and DIRECT. These results show that DCA already performs well for base-pair prediction. In contrast, DIRECT improves contacts involving tertiary interactions are improved. There are significant increases of 3~8 intra stem-stem contacts correctly predicted for 1Y26, 2GIS, 3OWI, and 3IRW. The intra stem-stem contacts indicate more bending or twisting contacts in these RNA structures. A more pronounced effect can be observed for the other three types of contacts (loop-loop, loop-stem, and inter stem-stem) predictions. In particular, contacts involving loop regions are more accurately predicted. The results show that DIRECT predicts better tertiary fold.

### DIRECT identifies more native contacts

**Fig. 2:**
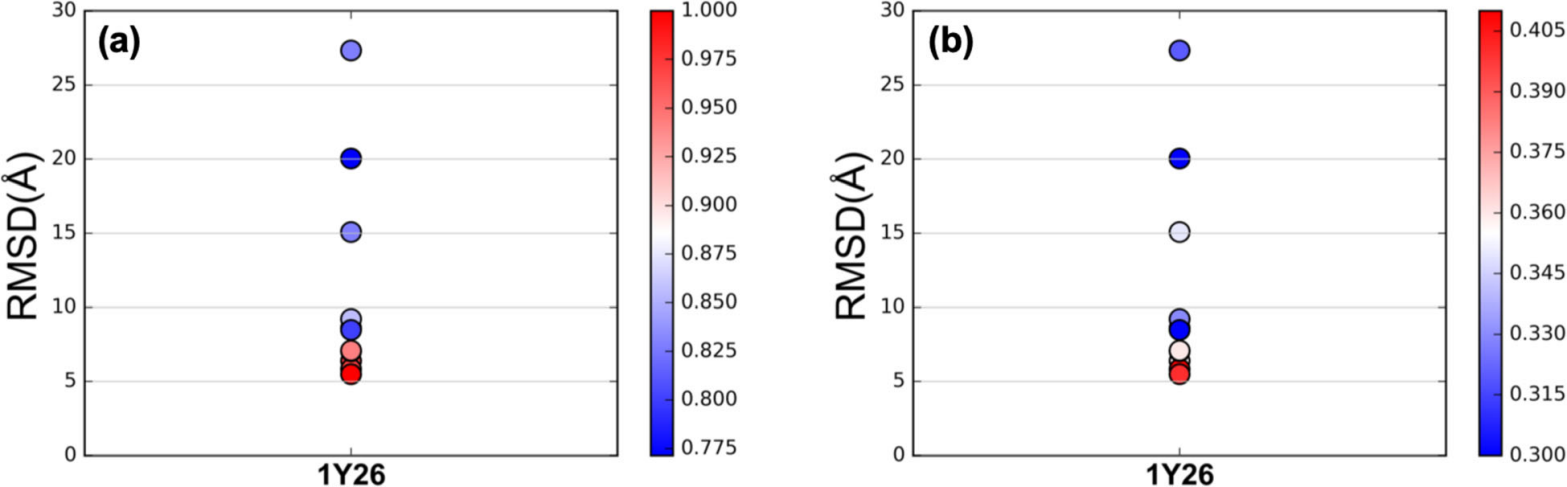
The contact and RMSD distributions in different RNA structures. The 11 structures are generated by 3dRNA, RNAcomposer, simRNA, and Vfold3D. **a** shows the correlation between native contacts and RMSDs. The predicted structure with the lowest RMSD contains 35 native contacts (100%) while the predicted structure with the largest RMSD contains 29 native contacts (83%). The color in (**b)** is the percentage of DIRECT predicted contacts out of the top 100 predicted contacts (RC/PC). The predicted structure with the lowest RMSD contains 40 predicted contacts (40%) while the predicted structure with the largest RMSD contains 32 predicted contacts (32%). The results suggest that DIRECT is able to identify more native contacts than non-native models with large RMSDs

To test if DIRECT is able to identify more native RNA contacts, we ran 4 popular RNA tertiary structure prediction programs (3dRNA, RNAcomposer, simRNA, and Vfold3D) on a given riboswitch to build a number of tertiary structures and evaluated the percentage of top contacts by DIRECT that were actually retained as the structure deviates from the native one. The results of riboswitch 1Y26 are shown in Fig. 2. All other riboswitch tests can be downloaded from our website. We analyzed the Predicted Contacts based on DIRECT prediction (PC), Native Contacts in PC (NC), and RNA Contacts based on predicted structures (RC). Figure 2a shows the correlation between native contacts and RMSDs. The all-atom root-mean-square deviation (RMSD) is measured against the true native structure. The color in Fig. 2a is the percentage of native contacts identified by DIRECT out of top 100 predicted contacts (RC/NC). The predicted structure with the lowest RMSD contains 35 native contacts (100%) while the predicted structure with the largest RMSD contains 29 native contacts (83%). The results show that the native-like structures have much more identified native contacts than the structures with large RMSD values. In addition, we tested the correlation between predicted contacts based on DIRECT prediction and RMSDs if we do not know the native structure. The color in Fig. 2b is the percentage of DIRECT predicted contacts out of the top 100 predicted contacts (RC/PC). The predicted structure with the lowest RMSD contains 40 predicted contacts (40%) while the predicted structure with the largest RMSD contains 32 predicted contacts (32%). The results also show that native-like structures have much more predicted contacts by DIRECT. All results suggest that DIRECT is able to identify more native contacts that define the true RNA tertiary structure.

### DIRECT improves RNA contact predictions using non-redundant RNA training sets

We then used another three non-redundant RNA training sets to evaluate the prediction accuracy of DIRECT. The non-redundant RNA training set 2 contains all the representative high-quality structures of 147 classes with length from 50 nt to 120 nt. The homology sequence or structure between training and testing sets were removed to ensure that RNAs in the training and testing sets have no sequence and structural overlap. As shown in Additional file 1: Figure S3, DIRECT is 21%~ 95% and − 4%~ 60% more precise (positive predictive value) than mfDCA and plmDCA, respectively, for 1Y26, 2GDI, 2GIS, 3IRW, 3OWI, and 3VRS predictions. Taken together, these results suggest that DIRECT is able to improve RNA contact predictions by learning structure template from more known structures as in the RNA non-redundant training set.

Another two non-redundant RNA training sets are as follows: (1) non-redundant RNAs with length from 50 nt to 120 nt without any riboswitch structure (training set 3 in Methods Section), and (2) all non-redundant RNAs without any riboswitch structure (training set 4 in Method Section). The results showed an average accuracy increase of 15 and 4% compared to mfDCA and plmDCA using training set 3, and 7 and 11% using the training set 4. The predictions clearly indicate that there are indeed generic and useful RNA structural features discerned by DIRECT that can improve contact prediction for a specific class of RNA.

### DIRECT achieves reliable prediction of conserved contacts

**Fig. 3:**
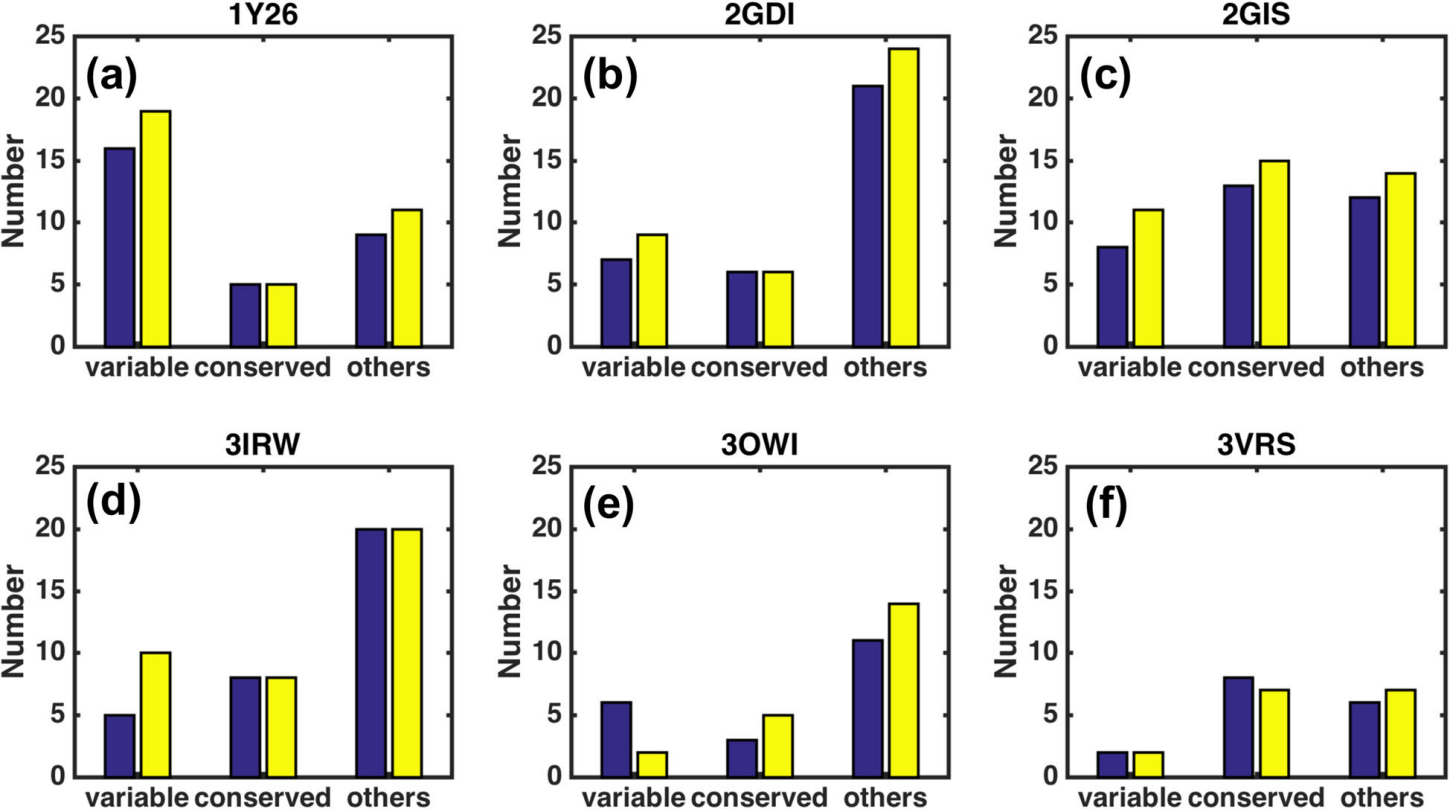
The number of correctly predicted contacts according to the conservation grades of the two nucleotides involved in the contact for all tested riboswitch RNAs. The contacts are divided into variable type (both nucleotides with conservation-grade 1–3), conserved type (both nucleotides with conservation-grade 7–9), and others, respectively. **a**, **b**, **c**, **d**, **e**, and **f** The performance of DIRECT (yellow) in comparison to DCA (blue) is considerably better for the variable contacts and only slightly improved for the conserved contacts

The hypothesis of direct coupling analysis stipulates that co-evolving nucleotides in an RNA molecule may form intra-molecular contacts to support its structure and function. DCA thus aims to disentangle the direct pairwise couplings from indirect correlations of the sequence variations. While highly conserved contacts are critical for RNA structural stability and function, their detection by DCA may be difficult due to insufficient information on variations. To examine if DIRECT can improve the prediction in this case, we divided nucleotides into different types based on their conservation scores calculated by the ConSurf program [47]. The continuous conservation scores are first divided into a discrete scale of 9 grades and the predicted contacts are then classified into three categories: variable contacts (both nucleotides in grade 1–3), conserved contacts (both nucleotides in grade 7–9), and other contacts. As shown in Fig. 3, DIRECT improves the prediction for variable contacts in 1Y26, 2GIS, and 3IRW as well as other contacts in 1Y26, 2GDI, 2GIS, and 3OWI. Although slight improvements observed, it is clear that additional information beyond sequence variation and the structural template is required to achieve a reliable prediction for conserved contacts.

## Discussion

Previous research suggests the number of sequences should be more than three times the length of the molecule for reliable contact prediction [28]. However, many RNA families do not satisfy this condition. While loosening the criterion for homology may result in more sequences, this approach inevitably leads to low accuracy in contact prediction. It remains challenging to extract evolutionary information from an insufficient number of sequences. To check if DIRECT can address the issue of insufficient sequences, we performed contact prediction on 5 target riboswitches using only 50 randomly chosen sequences. The lengths of the 5 RNAs range from 52 to 92 nucleotides and already exceed 50, the number of sequences used. The results in Additional file 1: Table S4 show that DIRECT outperforms DCA with an average increase of 12% in prediction precision suggesting that DIRECT can improve predictions even when the number of homologous sequences is insufficient.

To investigate the predictive accuracy on different structural templates, we incorporated a Restricted Boltzmann Machine (RBM) to augment the information on sequence co-variations with four different training sets in contact inference. The contacts learned by Restricted Boltzmann Machine fall mainly into two categories (Additional file 1: Figure S4). One is about the long-range contacts of loop-loop interactions, for example, the loop-loop contacts of A-riboswitch (PDB code: 1Y26), TPP riboswitch (PDB code: 2GDI), SAM-I riboswitch (PDB code: 2GIS), and c-di-GMP riboswitch (PDB code: 3IRW). The other one is about the contacts in junction regions. The contacts of glycine riboswitch (PDB code: 3OWI) and fluoride riboswitch (PDB code: 3VRS) define the junction orientations. Unlike local pattern recognition, the global indicator in terms of loop-loop or junctions contacts is more robust in capturing the folding topology of the entire structure beyond some particular parts. DIRECT is able to successfully identify the RNA contact with an average PPV around 0.6 in top 30 predicted contacts (Additional file 1: Figure S8).

## Conclusions

In summary, we developed a hybrid approach that incorporates a Restricted Boltzmann Machine (RBM) to augment the information on sequence co-variations with structural templates in contact inference. Our results demonstrated a 41 and 18% precision increase for RNA contact prediction in comparison to the mfDCA and plmDCA when structural templates are utilized. In fact, our approach establishes a straightforward framework that can incorporate any additional information, such as NMR spectroscopy data, by training a corresponding Restrictive Boltzmann Machine to further improve the prediction on RNA contacts.

## Methods

### Inference workflow

**Fig. 4:**
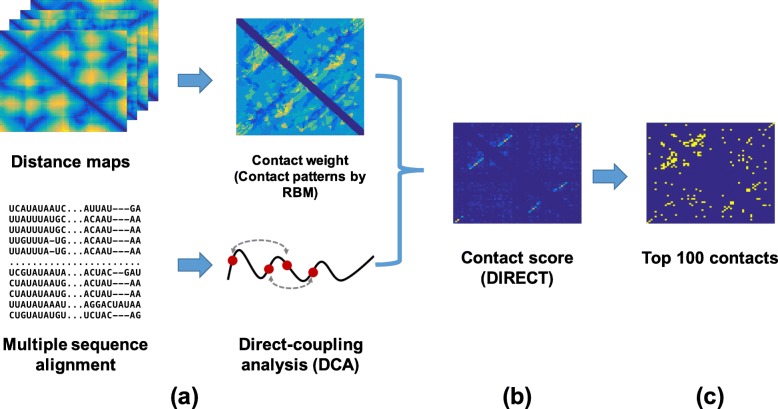
Basic workflow of DIRECT for RNA tertiary contact prediction. **a** The corresponding RNA multiple sequence alignment (MSA) is extracted from the Rfam database. The traditional direct-coupling analysis (DCA) predicts the tertiary contacts from sequence coevolution in MSA. **b** DIRECT then reweighs the contacts by using structural templates trained by Restricted Boltzmann Machine (RBM). **c** The reweighted contact prediction leads to better overall performance

DIRECT (Direct Information REweighted by Contact Templates) improves the prediction of tertiary contacts by using both sequence and structure information. Figure 4 illustrates the workflow of DIRECT. First, the corresponding RNA multiple sequence alignment (MSA) is extracted from Rfam database. Second, the traditional direct-coupling analysis (DCA) predicts the tertiary contacts from sequence coevolution in MSA. Third, contact weighs are calculated using structural templates trained by Restricted Boltzmann Machine (RBM). Then, DIRECT reweighs the mfDCA/plmDCA contact predictions. The inference framework consists of completely hierarchical modules and thus offers the flexibility to incorporate more sequences and structures that may become available in the future, as well as further improved DCA methods for enhanced performance.

### Restricted Boltzmann machine (RBM)

The Restricted Boltzmann Machine (RBM) is a graphical model for unsupervised learning that can extract features from the input data [48]. RBM has a visible layer and a hidden layer. The restriction is that units in the visible layer only interact with units from the hidden layer. This network structure leads to a factorized probability for observing a given configuration, which in turn further simplifies the learning process. The energy of an RBM is given by

1 $$ \mathrm{E}\left(\mathrm{v},\mathrm{h}|\mathrm{W},\mathrm{b},\mathrm{c}\right)=-{\mathrm{b}}^{\mathrm{T}}\mathrm{v}-{\mathrm{c}}^{\mathrm{T}}\mathrm{h}-{\mathrm{h}}^{\mathrm{T}}\mathrm{Wv} $$

where W is the connection weight matrix between visible v and hidden units h. b, c are bias units as offsets. The probability of having a given v, h is then

2 $$ \mathrm{p}\left(\mathrm{v},\mathrm{h}|\mathrm{W},\mathrm{b},\mathrm{c}\right)=\frac{1}{\mathrm{z}\left(\mathrm{W},\mathrm{b},\mathrm{c}\right)}{\mathrm{e}}^{-\mathrm{E}\left(\mathrm{v},\mathrm{h}|\mathrm{W},\mathrm{b},\mathrm{c}\right)} $$

3 $$ \mathrm{z}\left(\mathrm{W},\mathrm{b},\mathrm{c}\right)={\sum}_{\mathrm{v},\mathrm{h}}{\mathrm{e}}^{-\mathrm{E}\left(\mathrm{v},\mathrm{h}|\mathrm{W},\mathrm{b},\mathrm{c}\right)} $$

where z(W, b, c) is the partition function that sums up all possible v and h. The RBM is trained through stochastic gradient descent (SGD) on negative log-likelihood of the empirical data. L(W, c, b, T) is defined as the loss function, which we want to minimize during SGD:

4 $$ \mathrm{L}\left(\mathrm{W},\mathrm{c},\mathrm{b},\mathrm{T}\right)=-\frac{1}{\mathrm{N}}{\sum}_{\mathrm{v}\in \mathrm{T}}\log \mathrm{P}\left(\mathrm{v}|\mathrm{W},\mathrm{b},\mathrm{c}\right) $$

where P(v| W, b, c) is given by

5 $$ \mathrm{P}\left(\mathrm{v}|\mathrm{W},\mathrm{b},\mathrm{c}\right)={\sum}_{\mathrm{h}}\mathrm{p}\left(\mathrm{v},\mathrm{h}|\mathrm{W},\mathrm{b},\mathrm{c}\right) $$

T above is a set of samples from the empirical data. By minimizing the loss function, we can update the parameters W, b, c according to the equations below:

6 $$ \mathrm{W}=\mathrm{W}-\frac{\mathrm{\partial L}\left(\mathrm{W},\mathrm{b},\mathrm{c},\mathrm{T}\right)}{\mathrm{\partial W}} $$

7 $$ \mathrm{b}=\mathrm{b}-\frac{\mathrm{\partial L}\left(\mathrm{W},\mathrm{b},\mathrm{c},\mathrm{T}\right)}{\mathrm{\partial b}} $$

8 $$ \mathrm{c}=\mathrm{c}-\frac{\mathrm{\partial L}\left(\mathrm{W},\mathrm{b},\mathrm{c},\mathrm{T}\right)}{\mathrm{\partial c}} $$

### Contact definition and evaluation criteria

Two nucleotides are considered in contact if they contain a pair of heavy atoms, one from each nucleotide, less than a pre-defined cutoff [49–51]. Previous work indicated that 8 Å can serve as a reliable contact cutoff for RNA tertiary structural study [34, 35]. To compare DIRECT with earlier methods, we use the same reliable contact distance cutoff of 8 Å as in previous studies [34, 35]. A-form RNA rises 2.6 Å per base pair; the stacking interaction is thus small if the distance of two nucleotides is larger than 8 Å. Since adjacent nucleotides in a sequence are always in contact, we only consider contacts between nucleotides that are separated by more than four nucleotides in a sequence to measure tertiary contacts of interest. To evaluate the quality of a prediction, we compute the positive predictive value (PPV) as follows.

9 $$ PPV=\frac{\left| TP\right|}{\left| TP\right|+\left| FP\right|} $$

where TP (FP) denotes the true (false) positive and stands for the number of true (false) positives.

### Training and testing sets

Riboswitch is a regulatory portion of a messenger RNA. When binding with a small ligand, this regulatory segment will regulate the translation of the entire mRNA. In this study, we constructed four different training sets ranging from containing no homologous riboswitch with similar RNA size to strictly no riboswitch with all RNA sizes. This is to ensure that there is no data leakage between the training set and testing set so that the features captured by DIRECT are generic and useful RNA template patterns. Details of the datasets are as follows.

(1) RNA non-redundant training set 1. To generate a representative set of riboswitch families for our study, we systematically selected riboswitch families from the Rfam database. The ten representative riboswitches in the training set are shown in Additional file 1: Table S5. We analyzed the sequence identity value (calculated by CLUSTALW, http://www.genome.jp/tools-bin/clustalw) and structural similarity via RMSD value (calculated by PyMOL, www.pymol.org) between each pair of RNAs in the training set (Additional file 1: Table S6). The large values for sequence diversity and RMSD suggest that the RNAs in the training set share little similarity in sequence and structure.

(2) RNA non-redundant training set 2. We collected all the representative high-quality structures with resolution ≤3.0 Å of 147 classes of RNA 3D Hub non-redundant RNA set (version 3.21) with length from 50 nt to 120 nt [52]. RNA 3D Hub ensured that sequence identity between any two sequences is < 95%. It is noted that existing methods for RNA tertiary structure prediction (for example, RASP and 3dRNA) also used a sequence identity of 95% to reduce redundancy in training set [14, 15, 53]. RNAs that share sequence or structure homology in training and testing sets were removed from the training set. These steps ensure that structures in the training set and testing sets have a similar size but no sequence and structural overlap.

(3) RNA non-redundant training set 3. We collected all the representative high-quality structures in RNA non-redundant training set 2, then removed all riboswitch structures in this training set. These steps ensure no riboswitch structure in the training set.

(4) RNA non-redundant training set 4. Unlike RNA non-redundant training set 3 that collected similar size RNAs as a testing set (from 50 nt to 120 nt), we collected all the representative high-quality tertiary structures with resolution ≤3.0 Å of 1023 classes of RNA 3D Hub non-redundant RNA set (version 3.21). Then, we also removed all the riboswitch structures in this training set. These steps ensure no riboswitch structure in the training set. The lists of RNA non-redundant training set 3 and 4 can be downloaded from our website.

For the testing set, we used the published testing dataset including six riboswitches (Additional file 1: Table S1) [34].

### Weight of structural information learned by RBM for prediction of riboswitch

**Fig. 5:**
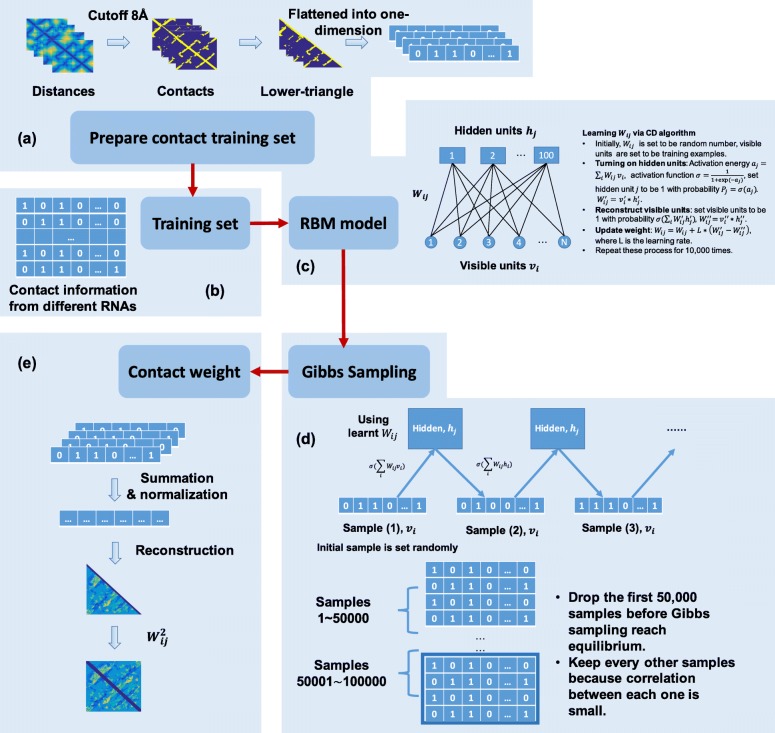
Further refined workflow for part of Fig. 4 on training a Restricted Boltzmann Machine (RBM) to detect contact patterns. Specific steps to extract the contact weights from RNA tertiary structure are as follows. **a** Prepare contact training set. A contact map of a given RNA is constructed from its nucleotide-nucleotide distance matrix. Two nucleotides are considered in contact if a pair of heavy atoms, one from each nucleotide, is less than 8 Å apart. The lower triangles of the contact map are maintained and then converted to a one-dimensional array as the input to RBM. **b** Training set. The training set consists of all contact maps of riboswitch structures but with the testing homologous riboswitch structure removed. **c** RBM model. Parameters in RBM are trained by the Contrastive Divergence (CD) algorithm. **d** Gibbs sampling. We run Gibbs sampling to generate new contact maps using RBM model. The last 50,000 samples are maintained for contact weight calculation. **e** Contact weight. The Gibbs sampling results are normalized into one contact matrix representing nucleotide-nucleotide contact weights for a typical riboswitch structure

The Restricted Boltzmann Machine (RBM) is used to extract the contact knowledge from riboswitch structures in the training set (Fig. 5).

Step 1: Prepare the training set (Fig. 5a and b). Riboswitch structures in the training set are converted into contact maps by applying the distance cutoff of 8 Å. The lengths of the testing riboswitches range from 52 to 94 nucleotides. For the convenience of integrating the templates of structural information, all distance maps are resized by linear interpolation into the same size of 100 × 100 pixels before applying the distance cutoff of 8 Å. Image resizing is widely used in deep learning communities to train a machine with fixed input of picture sizes. One of the popular architecture of convolutional neural networks, VGG-16, is trained with over 14 million images belonging to 1000 classes [54]. All images are resized into a size of 224 × 244 × 3 (RGB image) before being fed into the machine. VGG-16 achieves 70.5% accuracy for top 1 prediction and 90% accuracy for top 5 predictions. Following a similar consideration and given that the riboswitches in our training set have varying lengths of 54 to 94 nucleotides, we also resized distance maps into a fixed size of 100 × 100 and then converted it into a contact map using a cutoff of 8 Å. The resizing done by the linear interpolation will keep the spatial information invariant between nucleotides. For example, the distance between nucleotide 1 and 4 in an RNA with 50 nucleotides will stay the same between position 1 and 8 after we resize the distance matrix into 100 × 100. The contact patterns are almost identical between original and resized ones (Additional file 1: Figure S5) since one is a coarse-grained version of the other. These results show that the global features and local patterns are kept during the resizing. To remove the overlap between the training set and testing set, we exclude all homologous training structures with respect to the riboswitch structure in the testing set for each prediction. To be more precise for this blind test, when predicting each of the six riboswitches in the test set, the targeting riboswitch and all its homologs are removed from the training set. RBM learned six different weights of structural information for the six riboswitches. We converted the lower triangle contact maps into a one-dimensional array with one channel per contact (as 1) or non-contact (as 0). The elements of this one-dimensional array will be fed into the visible units of RBM. Thus, in our six different tests, the number of visible units of RBM is determined by the size of the contact map (or in other words, the number of nucleotides in the riboswitch). The length of our testing riboswitches is between 52 and 94 nucleotides. Moreover, the optimal number of hidden units is determined to be 100 via a grid search (Additional file 1: Figure S5).

Step 2: Learning the shared weights (Fig. 5c). Training the RBM efficiently by stochastic gradient descent (SGD) involves an algorithm called Contrastive-Divergence (CD) invented by Hinton [55]. In this study, we use a typical learning rate of 0.1 and epochs of 10,000 during RBM training.

Step 3: Gibbs sampling (Fig. 5d). After RBM is trained from the structures of existing riboswitch RNA, we generated 10,000 new structures and kept the last 5000 structures to model the equilibrium that represents RBM’s belief for the most common structure of riboswitches. What the RBM learned in the previous step is the hidden connections between hidden representations and contact patterns (visible representations). Gibbs sampling method is the widely used approach to get samples from an energy-based model. We turned the model into a generative mode to produce visible contact patterns through a Gibbs sampling process. To get the unbiased samples from the model we learned in the previous step, we need to run Gibbs sampling for a long time for convergence. 10,000 samples generated by the model at this stage are converged.

Step 4: Contact weight learned by RBM (Fig. 5e). We counted the contact frequency for each nucleotide among these 5000 structures and took this frequency as the final weight matrix learned by RBM on the structure information of the riboswitch.

### Direct coupling analysis

The direct coupling analysis (DCA) is performed to infer the interacting nucleotides from sequence coevolution across different species [22, 35, 56]. We first removed the sequences with gaps of more than 50% in multiple sequence alignment (MSA) and then calculated the amino acid frequencies for single-nucleotide and a pair of nucleotides. The direct couplings that indicate the interaction strength between two sites are defined as

10 $$ {DI}_{ij}={\sum}_{AB}{P}_{ij}^d\left(A,B\right)\mathit{\ln}\frac{P_{ij}^d\left(A,B\right)}{f_i(A){f}_j(B)} $$

with the help of an isolated two-site model

11 $$ {P}_{ij}^d\left(A,B\right)=\mathit{\exp}\left\{{e}_{ij}\left(A,B\right)+\tilde{h}_{i}(A)+\tilde{h}_{j}(B)\right\}/{Z}_{ij} $$

$\tilde{h}_{i}(A)$and $\tilde{h}_{j}(B)$ are defined by the empirical single-nucleotide frequency ${f}_i(A)={\sum}_B{P}_{ij}^d\left(A,B\right)$ and ${f}_j(B)={\sum}_A{P}_{ij}^d\left(A,B\right)$. Mean-field DCA (mfDCA) is done by a simple mean-field approximation, see Morcos et al. (Morcos, et al., 2011) for details. Ekeberg also proposes a method called plmDCA using pseudo-likelihood maximization for inferring direct coupling [37, 57]. We used the downloaded versions of mfDCA and plmDCA algorithms. The mfDCA was downloaded from http://dca.rice.edu/portal/dca/download. The plmDCA was downloaded from the Marks lab at Harvard Medical School (http://evfold.org/evfold-web/code.do).

### Direct information scores reweighted by structural contact frequency

The final contact prediction is DI scores reweighted by structural information learned by RBM with better contact prediction accuracy.

12 $$ DIRECT= DI\times {W}^2 $$

where DI is the direct information by direct coupling analysis, W is RBM-based structural contact frequency. Among the different powers of W considered (up to the 4th power), we finally selected the 2nd power of W as in Eq. (12) to balance the contributions from both patterns of sequence evolution and RBM-based structural contact frequency.

### Tertiary structure prediction

We predicted RNA tertiary structures using 3dRNA, RNAcomposer, simRNA and Vfold3D [11, 14, 15, 17, 58]. For each RNA structure prediction, we used the corresponding sequence and secondary structure on the RNA structure modeling servers. All tertiary structures are predicted automatically.

### Regularization

Regularization is a strategy that aims to reduce the generalization errors [59]. Most machine learning methods add restrictions on the parameters. For example, L1 and L2 regularization are adding a cost function that penalizes high-value weights to prevent overfitting. The weight of RBM is a matrix of 4951 × 101 (connecting the 4950 visible units and 100 hidden units, the one extra unit on each side is the bias unit). Although we did not implement the regularization in our model training, the obtained weights shown in Additional file 1: Figure S7 did not take extreme values associated with overfitting.

## Supplementary information

**Additional file 1.** Supplementary material, including all supplementary figures and supplementary tables.

## Data Availability

The codes and dataset are available at https://zhaolab.com.cn/DIRECT/.

## Abbreviations

CD: Contrastive-Divergence
DCA: Direct coupling analysis
DI: Direct information
DIRECT: Direct Information REweighted by Contact Templates
FP: False positive
inter-molecule: Across the homodimer interface
intra-molecule: Interact within the same molecule
mfDCA: Mean-field direct coupling analysis
MSA: Multiple sequence alignment
NC: Native Contacts
NMR: Nuclear Magnetic Resonance
PC: Predicted Contacts
plmDCA: Pseudo-likelihood maximizes direct coupling analysis
PPV: Positive predictive value
RBM: Restricted Boltzmann Machine
RC: RNA Contacts
RMSD: Root-mean-square deviation
SGD: Stochastic gradient descent
TP: True positive
