# Supplementary material for: DIRECT: RNA contact predictions by integrating structural patterns
Source: BMC Bioinformatics. 2019 Oct 15;20:497. doi: 10.1186/s12859-019-3099-4 (PMC6794908; doi:10.1186/s12859-019-3099-4)
Supplement: Supplementary file 1 — Additional file 1. Supplementary material, including all supplementary figures and supplementary tables. [file 12859_2019_3099_MOESM1_ESM.pdf]

Supplementary material:

DIRECT: RNA Contact Predictions by Integrating Structural Patterns

Yiren Jian[1,2+], Xiaonan Wang[1+], Jiadi Qiu[1], Huiwen Wang[1], Zhichao Liu[2], Yunjie Zhao[1*], Chen Zeng[2*]

Author affiliation:

[1]Institute of Biophysics and Department of Physics, Central China Normal University, Wuhan 430079, China

[2]Department of Physics, The George Washington University, Washington, DC 20052, USA

[+]These authors contributed equally to the paper as first authors

*Corresponding author

Correspondence:

Yunjie Zhao Email: yjzhaowh@mail.ccnu.edu.cn

Chen Zeng Email: chenz@gwu.edu

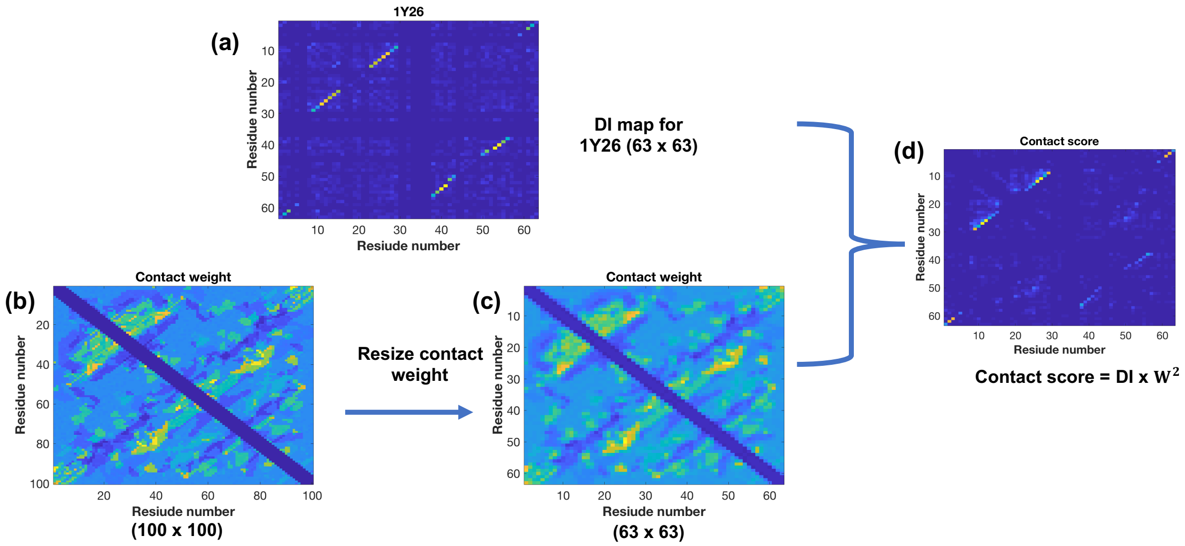

Figure S1: The workflow for DIRECT making contact score. **(a)** The DI score for 1Y26 (with 63 nucleotides). The DI map is a picture of size 63 x 63. **(b)** A contact weight of size 100 x 100 that is trained by RBM. **(c)** To incorporate the contact weight onto the DI map for 1Y26, we resize the contact weight from 100 x 100 to 63 x 63. **(d)** The final contact score is DI x W^2, where DI is DI score and W is contact weight.

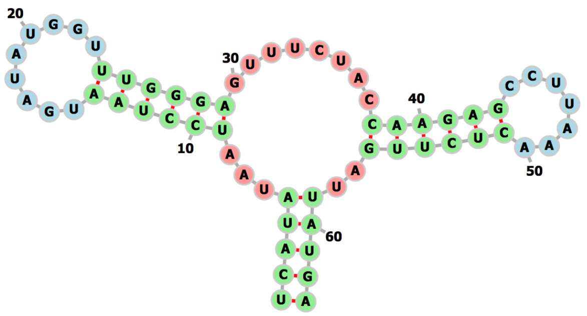

(a) 1Y26 RF00167

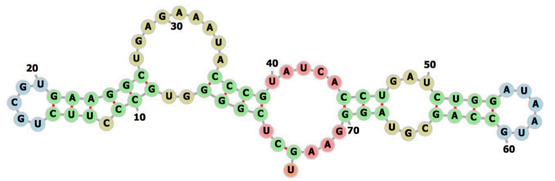

(b) 2GDI RF00059

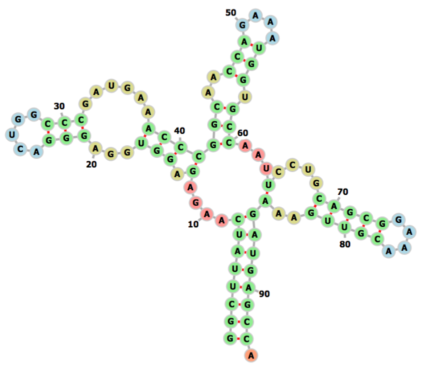

(c) 2GIS RF00162

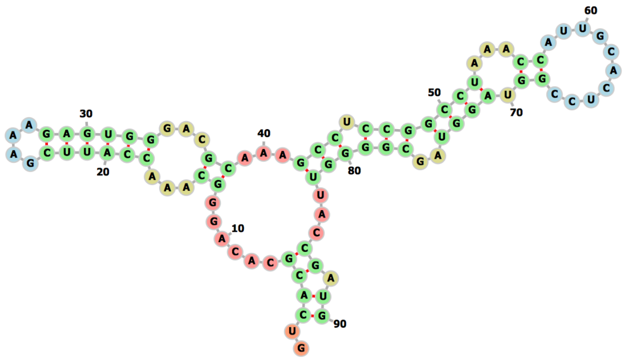

(d) 3IRW RF01051

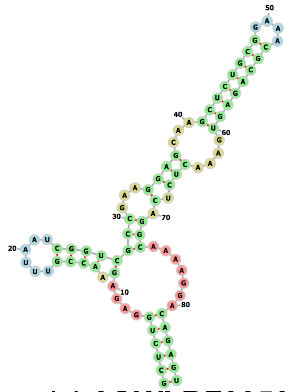

(e) 3OWI RF00504

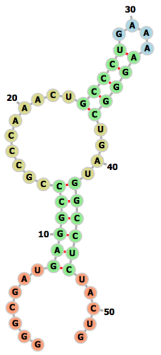

(f) 3VRS RF01734

Figure S2: The secondary structures of RNAs in the testing set. The green nucleotides are for base pairs, blue for hairpin loops, yellow for internal/bulge loops, and red for junctions or exterior loop. The secondary structures are generated by Forna program.

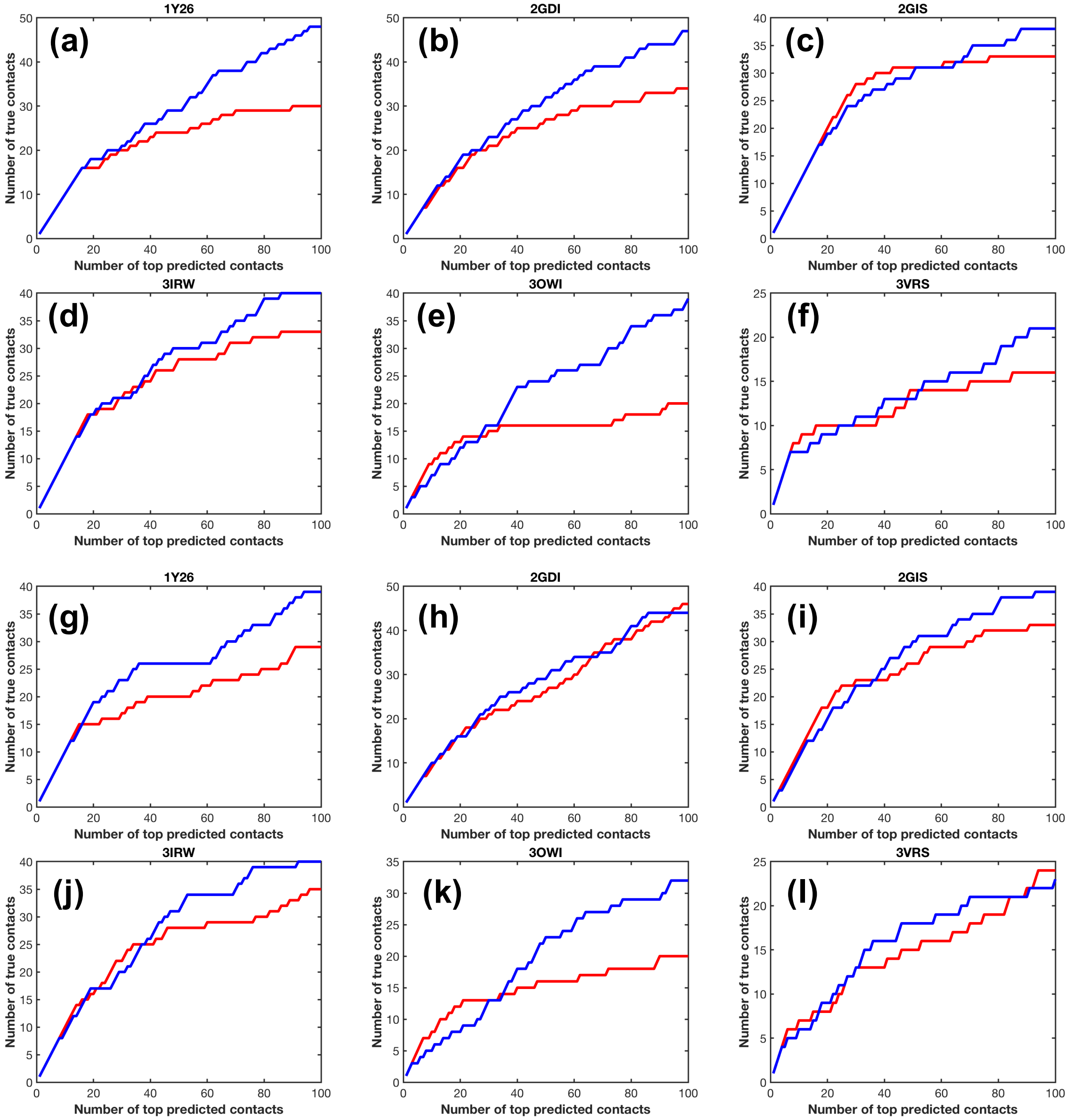

Figure S3: DIRECT vs. DCA. Accuracy of nucleotide-nucleotide contact prediction for all six RNAs in the testing set. **(a, b, c, d, e, f)** Comparison between DIRECT and mfDCA. The number of true contacts among a number of top predicted contacts is shown for each of the six RNAs. DIRECT (blue lines) achieves 41% higher true positive on average than mfDCA (red lines) for true contacts among the top 100 predicted contacts. **(g, h, I, j, k, l)** Comparison between DIRECT and plmDCA. DIRECT (blue lines) achieves 18% higher true positive on average than plmDCA (red lines) for true contacts among the top 100 predicted contacts.

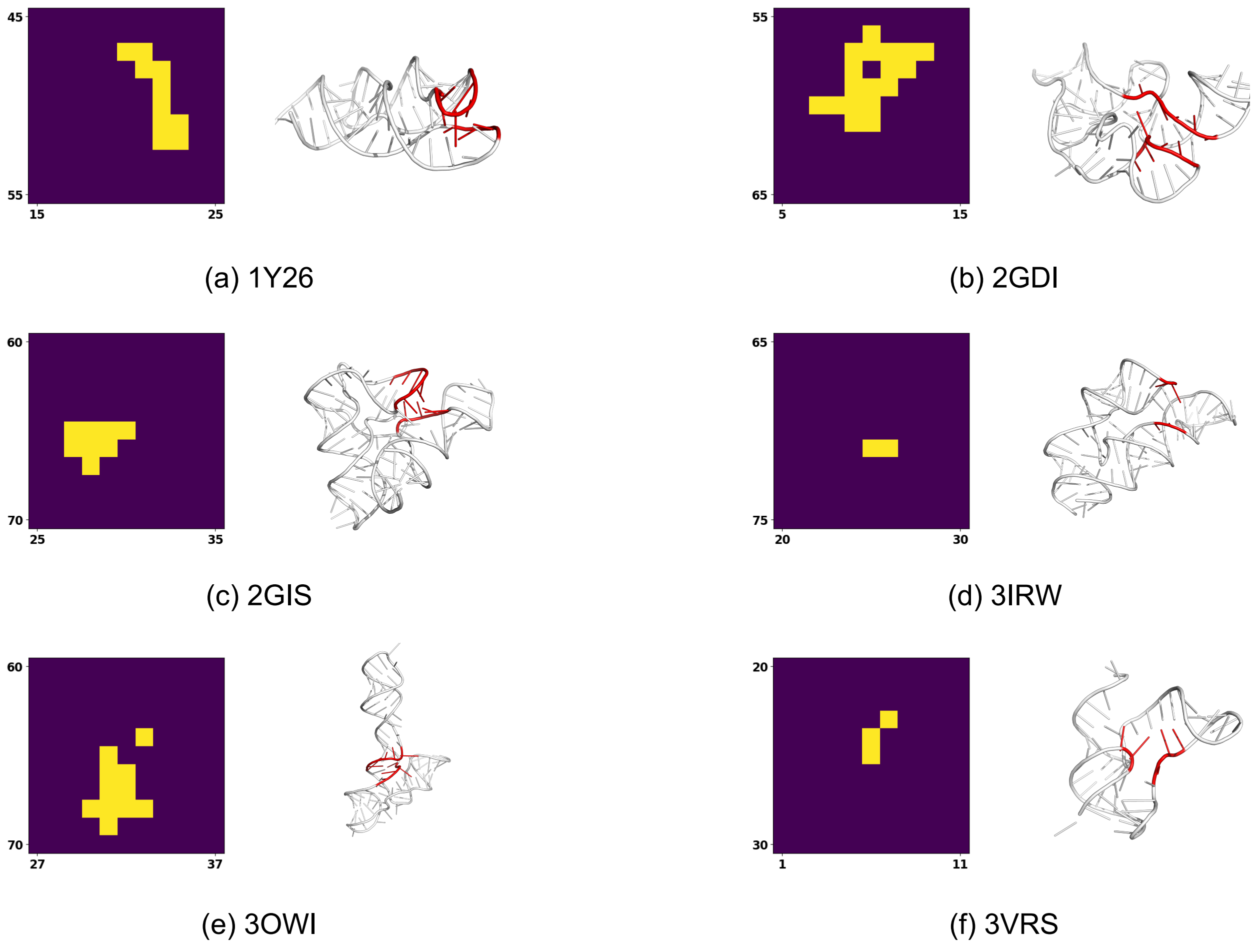

Figure S4: Contact patterns learned by contact weight of Restricted Boltzmann Machine and the corresponding structural motifs. The overlaps of real contacts and contact weights learned by RBM are shown on the left of (a), (b), (c), (d), (e) and (f). The corresponding structural motifs are highlighted in red showing on the right.

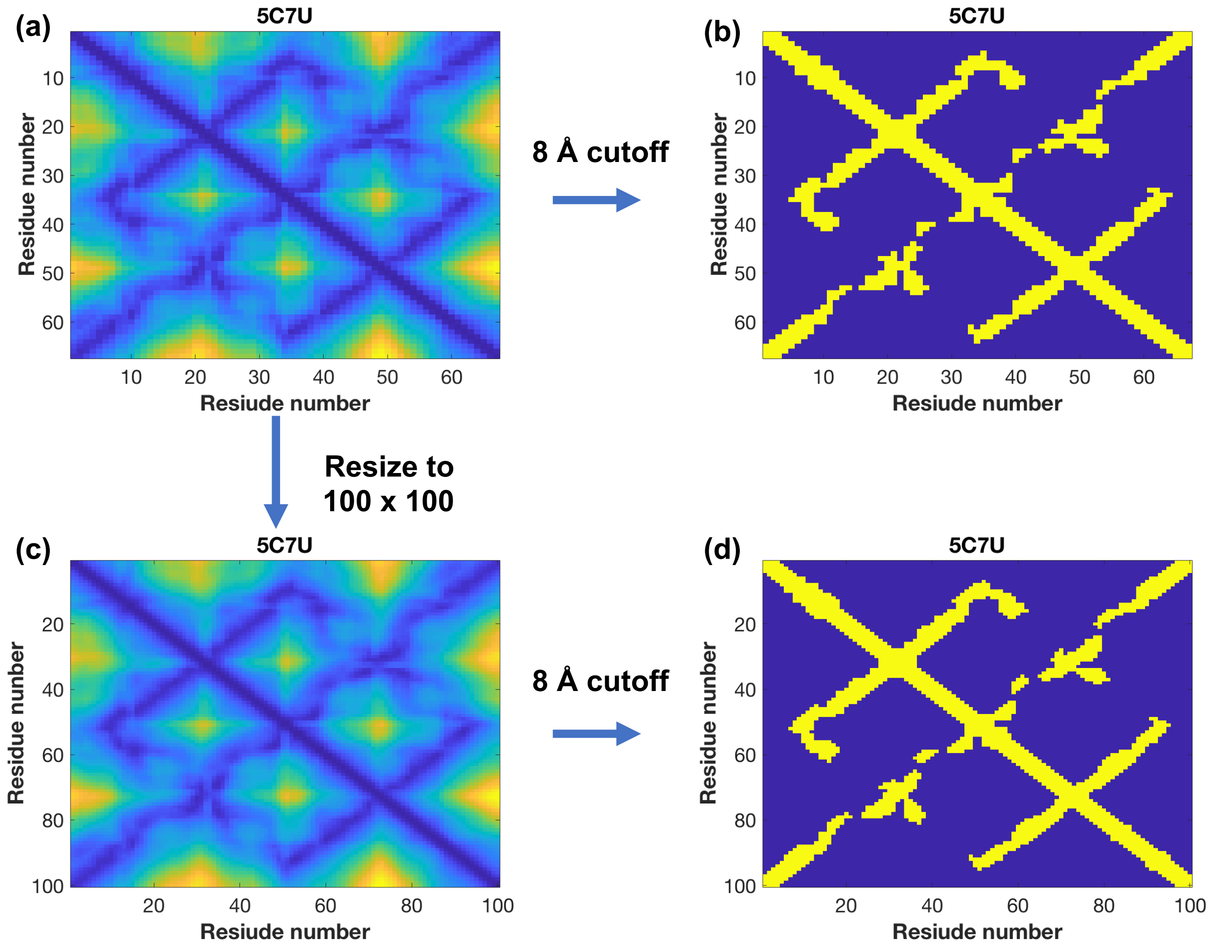

Figure S5: Distance map resizing and contact map generating. **(a)** A distance map for riboswitch 5c7u with 67 nucleotides is represented as an image of size 67 x 67. **(b)** By applying a cutoff of 8 Å, a contact map of size 67 x 67 is generated for 5c7u. **(c)** In order to train a Boltzmann machine for riboswitches of different number of nucleotides, we resize the distance map into a fixed size of 100 x 100. The example shown here (5c7u) is transformed from a 67 x 67 to a 100 x 100 distance map. The resizing algorithm for image uses bicubic interpolation. **(d)** Then we apply a cutoff of 8 Å on the resized distance map to generate the contact map used for machine training. By comparing (a) (c) and (b) (d), we find that the resizing algorithm can keep most the contact patterns in the original contact map.

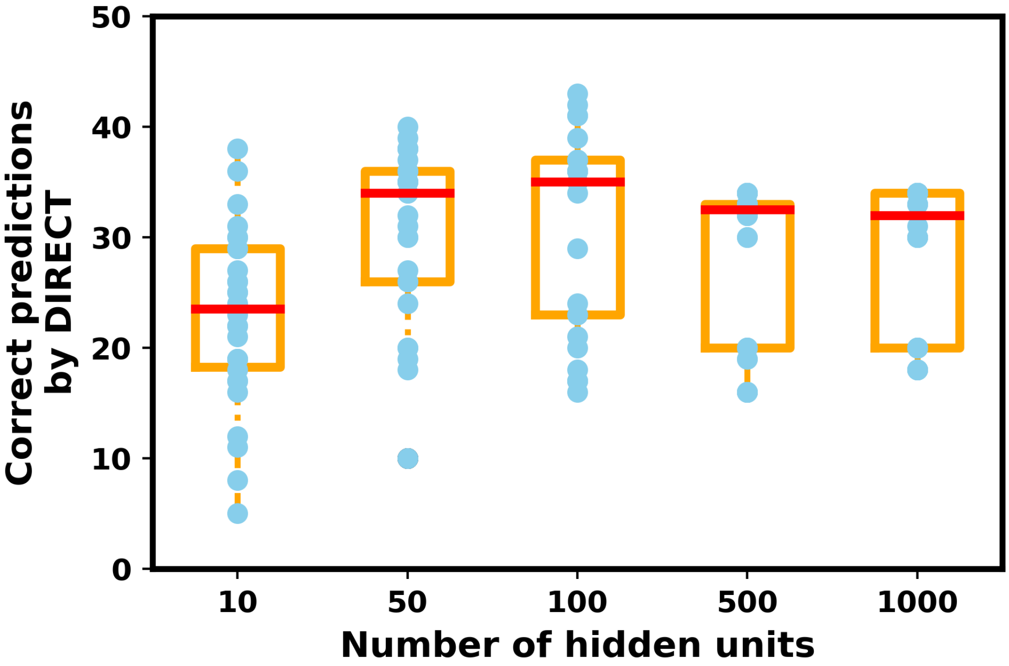

Figure S6: The optimal hidden units. We go through a grid search of 5-fold validation to test different number of hidden units such as 10, 50, 100, 500 and 1000. The best median and highest number of correct predictions by DIRECT is obtained by choosing 100 hidden units in RBM. RBM with a small number of hidden units of 10 may not be sufficient to cover the entire data space. The under-fitting in the training due to the small number of hidden units leads to some poor predictions in testing. On the other hand, overfitting by RBM with such large number of hidden units as 500 or 1000 leads to little or no improvement over traditional DCA. We thus choose 100 as the optimal number of hidden units for our RBM.

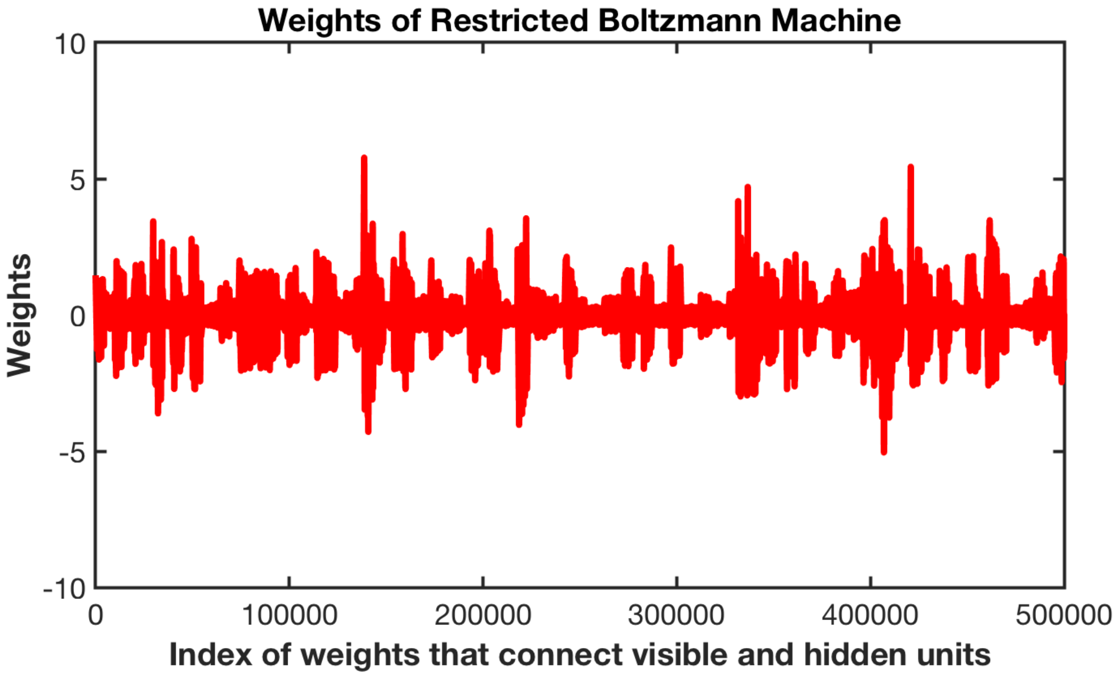

Figure S7: The weights of Restricted Boltzmann Machine. Though we do not implement the regularization term in training the weights that connect visible and hidden units, the final weights obtained nonetheless do not show extreme values associated with overfitting.

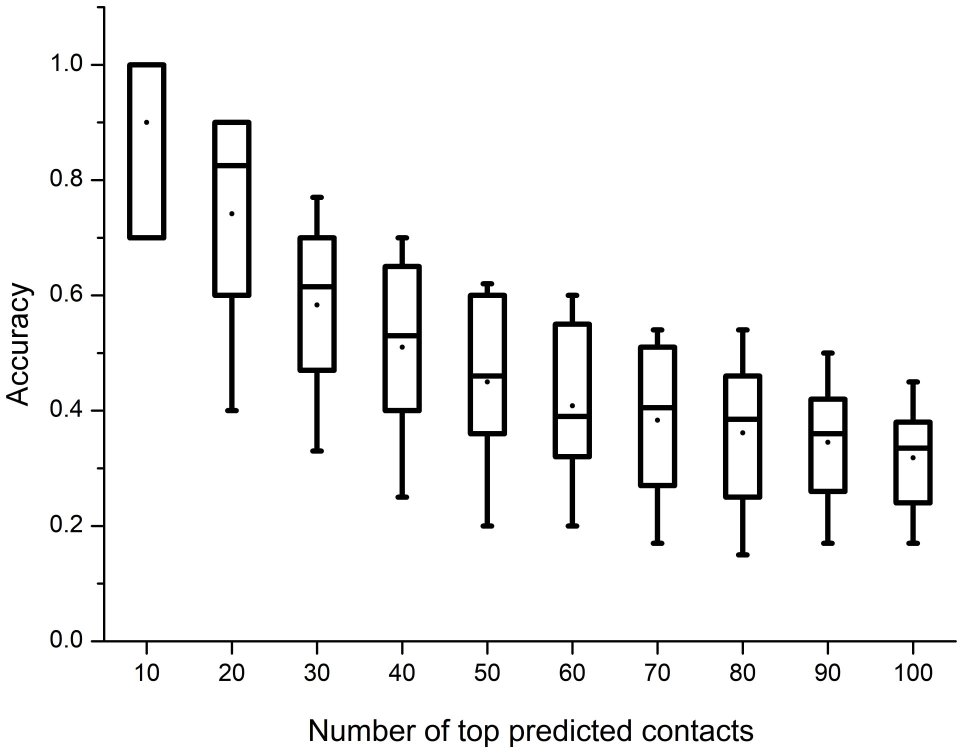

Figure S8: The contact prediction accuracy of DIRECT. DIRECT is able to successfully identify the RNA contact with an average PPV around 0.6 in top 30 predicted contacts.

Table S1: Selected riboswitches in the testing set.

PDB	Riboswitch type	Method	Resolution (Å)	Chain	Length	Rfam
1Y26	Adenine	X-RAY	2.10	X	63	RF00167
2GDI	TPP	X-RAY	2.05	X	75	RF00059
2GIS	SAM-I	X-RAY	2.90	A	94	RF00162
3IRW	c-di-GMP	X-RAY	2.70	R	90	RF01051
3OWI	Glycine	X-RAY	2.85	A	86	RF00504
3VRS	Fluoride	X-RAY	2.60	A	52	RF01734

The columns are PDB ID, riboswitch type, experimental method, structural resolution, selected chain, sequence length, and Rfam ID, respectively.

Table S2: Number of correctly predicted contacts among the top 100 predictions further grouped into three categories according to their base-pair (bp) separation in sequence for short (5~12bps), medium (13~24bps), and long (>24bps) ranges, respectively.

PDB	Ranges	mfDCA	DIRECT[1]	plmDCA	DIRECT[2]
	5~12	10	11	10	12
1Y26	13~24	12	15	11	13
	24+	8	9	8	10
	5~12	10	13	13	11
2GDI	13~24	6	5	12	11
	24+	18	21	21	19
	5~12	10	11	10	11
2GIS	13~24	8	9	7	8
	24+	15	20	16	23
	5~12	4	3	5	3
3OWI	13~24	5	1	3	1
	24+	11	17	12	24
	5~12	6	7	6	7
3IRW	13~24	6	6	7	7
	24+	21	25	22	23
	5~12	8	9	9	9
3VRS	13~24	1	1	2	1
	24+	7	6	13	11

Results obtained from different methods, mfDCA, DIRECT[1](mfDCA+RBM), plmDCA and DIRECT[2](plmDCA+RBM) are provided for comparison.

Table S3: Number of correctly predicted contacts among top 100 predictions for different classes of RNA.

PDB	Interactions	mfDCA	DIRECT[1]	plmDCA	DIRECT[2]
	Base pairs	15	15	15	15
	stem-loop	5	5	5	6
1Y26	loop-loop	2	2	3	7
	stem-stem(intra)	6	10	5	6
	stem-stem(inter)	2	3	1	1
	Base pairs	12	12	12	12
2GDI	stem-loop	12	15	17	15
	loop-loop	6	8	10	7
	stem-stem(intra)	3	3	6	5

	stem-stem(inter)	1	1	1	2
2GIS	Base pairs	21	21	21	20
	stem-loop	1	4	3	5
	loop-loop	9	5	8	5
	stem-stem(intra)	2	9	1	9
	stem-stem(inter)	0	1	0	3
3OWI	Base pairs	13	10	13	11
	stem-loop	3	1	2	3
	loop-loop	1	3	2	4
	stem-stem(intra)	3	6	3	10
	stem-stem(inter)	0	1	0	0
3IRW	Base pairs	16	16	17	17
	stem-loop	6	7	6	5
	loop-loop	2	2	4	3
	stem-stem(intra)	8	12	8	11
	stem-stem(inter)	1	1	0	1
3VRS	Base pairs	6	6	7	6
	stem-loop	1	1	5	4
	loop-loop	7	7	10	9
	stem-stem(intra)	2	2	2	2
	stem-stem(inter)	0	0	0	0

Results obtained from different methods, mfDCA, DIRECT[1](mfDCA+RBM), plmDCA and DIRECT[2](plmDCA+RBM) are provided for comparison.

Table S4: Comparison for the number of correctly predicted contacts among the top 100 predictions of DCA and DIRECT in a simulated dataset by randomly selecting only 50 sequences.

PDB	DCA	DIRECT
1Y26	27	33
2GDI	27	29
2GIS	22	23
3OWI	20	21
3IRW	22	29
3VRS	19	18

This examines the model performance when available sequences are insufficient.

Table S5: Selected riboswitches in the training set.

PDB	Method	Resolution (Å)	Chain	Length
2CKY	X-RAY	2.90	A	77
2MIY	NMR		A	59
3DJ2	X-RAY	2.50	A	174
3F2Q	X-RAY	2.95	X	112
3IQR	X-RAY	2.55	A	94
3MXH	X-RAY	2.30	R	92
3OWW	X-RAY	2.80	A	88
4EN5	X-RAY	2.96	A	52
4GMA	X-RAY	3.94	Z	210
5C7U	X-RAY	3.05	B	67

The columns are PDB ID, experimental method, structural resolution, selected chain, and sequence length, respectively.

Table S6: The sequence diversity (highlight in blue) and RMSD (highlight in green) values between each RNAs in the training set.

PDB	2cky	2miy	3dj2	3f2q	3iqr	3mxh	3oww	4en5	4gma	5c7u
**2cky**		18.18	22.78	19.04	22.36	23.59	23.28	17.97	26.71	14.04
**2miy**	27.12		23.35	19.45	18.51	24.32	24.89	18.68	25.82	19.53
**3dj2**	27.27	28.81		32.91	29.17	28.35	35.68	25.14	29.85	26.56
**3f2q**	25.97	28.81	27.68		22.50	26.08	26.71	20.22	26.06	22.54
**3iqr**	29.87	32.20	27.66	31.91		24.17	22.73	12.80	27.30	18.60
**3mxh**	20.78	25.42	32.61	18.48	27.17		25.03	22.44	28.33	20.74
**3oww**	23.38	22.03	29.55	27.27	25.00	22.73		25.63	27.40	26.91
**4en5**	28.85	21.15	38.46	26.92	34.62	30.77	28.85		24.36	17.41
**4gma**	29.87	30.51	24.14	25.00	30.85	32.61	26.14	42.31		25.54
**5c7u**	20.90	22.03	31.34	28.36	28.36	32.84	25.37	26.92	23.88	

The large sequence diversity and RMSD values suggest that the RNAs in the training set share little similarity.

Table S7: The RMSD values and contact information for predicted RNA tertiary structures.

PDB	Method	RMSD(Å)	Contact
1y26	3dRNA	6.39	25-50
1y26	3dRNA	5.83	25-50
1y26	3dRNA	7.04	25-50
1y26	3dRNA	8.61	no
1y26	3dRNA	5.49	25-50
1y26	RNAcomposer	27.32	no
1y26	simRNA	9.90	no
1y26	simRNA	9.22	no
1y26	simRNA	8.48	25-50
1y26	simRNA	15.08	no
1y26	simRNA	20.03	no
1y26	Vfold3D	7.08	25-50
2gdi	3dRNA	21.85	no
2gdi	3dRNA	16.77	no
2gdi	3dRNA	22.67	no
2gdi	3dRNA	20.65	no
2gdi	3dRNA	8.56	8-60
2gdi	RNAcomposer	9.30	no
2gdi	simRNA	24.06	no
2gdi	simRNA	20.31	no

2gdi	simRNA	27.35	no
2gdi	simRNA	21.35	no
2gdi	simRNA	21.28	no
2gis	3dRNA	4.04	15-42
2gis	3dRNA	4.03	15-42
2gis	3dRNA	5.33	15-42
2gis	3dRNA	5.70	15-42
2gis	3dRNA	9.87	15-42
2gis	RNAcomposer	4.33	15-42
2gis	simRNA	22.20	no
2gis	simRNA	16.98	15-42
2gis	simRNA	21.35	no
2gis	simRNA	16.72	15-42
2gis	simRNA	17.93	15-42
3irw	3dRNA	9.10	7-89
3irw	3dRNA	4.22	7-89
3irw	3dRNA	6.05	7-89
3irw	3dRNA	5.20	7-89
3irw	3dRNA	9.89	7-89
3irw	RNAcomposer	5.70	7-89
3irw	simRNA	20.72	no
3irw	simRNA	21.69	no
3irw	simRNA	21.57	no
3irw	simRNA	20.44	no
3irw	simRNA	23.21	no
3irw	Vfold3D	1.89	7-89
3owi	3dRNA	2.63	13-73
3owi	3dRNA	5.21	13-73
3owi	3dRNA	6.08	13-73
3owi	3dRNA	6.08	13-73
3owi	3dRNA	9.64	13-73
3owi	RNAcomposer	6.42	13-73
3owi	simRNA	13.88	no
3owi	simRNA	17.40	no
3owi	simRNA	12.93	no
3owi	simRNA	17.18	no
3owi	simRNA	13.47	no
3owi	Vfold3D	3.97	13-73
3vrs	3dRNA	14.93	no
3vrs	3dRNA	15.61	16-22

3vrs	3dRNA	13.77	no
3vrs	3dRNA	16.02	no
3vrs	3dRNA	16.38	no
3vrs	RNAcomposer	13.20	16-22
3vrs	simRNA	16.83	no
3vrs	simRNA	10.36	16-22
3vrs	simRNA	16.86	no
3vrs	simRNA	12.26	16-22
3vrs	simRNA	12.76	no
3vrs	Vfold3D	15.17	no

Table S8. The non-redundant RNAs in the training set extracted from RNA 3D Hub.

#	Equivalence class	Representative	Resolution	Nts	Class members
1	NR_3.0_51824.1	5WTI|1|B (5WTI)	2.7 Å	115	(1) 5WTI|1|B
2	NR_3.0_53735.1	4QLM|1|A (4QLM)	2.7 Å	108	(2) 4QLM|1|A, 4QLN|1|A
3	NR_3.0_47203.1	3F2Q|1|X (3F2Q)	3.0 Å	107	(2) 3F2Q|1|X, 3F2T|1|X
4	NR_3.0_83641.1	4Y1M|1|B (4Y1M)	3.0 Å	107	(2) 4Y1M|1|B, 4Y1M|1|A
5	NR_3.0_27096.1	4WFL|1|A (4WFL)	2.5 Å	105	(1) 4WFL|1|A
6	NR_3.0_00143.1	4Y1J|1|A (4Y1J)	2.2 Å	100	(4) 4Y1J|1|A, 4Y1J|1|B, 4Y1I|1|A, 4Y1I|1|B
7	NR_3.0_20814.1	3SUX|1|X (3SUX)	2.9 Å	100	(2) 3SUX|1|X, 3SUH|1|X
8	NR_3.0_50424.1	5U30|1|B (5U30)	2.9 Å	99	(2) 5U30|1|B, 5U31|1|B
9	NR_3.0_37714.1	4RZD|1|A (4RZD)	2.8 Å	98	(1) 4RZD|1|A
10	NR_3.0_12869.1	4L81|1|A (4L81)	3.0 Å	96	(1) 4L81|1|A
11	NR_3.0_31222.1	5FJC|1|A (5FJC)	1.7 Å	96	(12) 5FJC|1|A, 5FK4|1|A, 4B5R|1|A, 5FK3|1|A, 5FK2|1|A, 5FKF|1|A, 5FK6|1|A, 5FK1|1|A, 5FKD|1|A, 5FKH|1|A, 5FKG|1|A, 5FKE|1|A
12	NR_3.0_77464.1	4AOB|1|A (4AOB)	3.0 Å	94	(2) 4AOB|1|A, 4AEB|1|A

13	NR_3.0_82809.4	2YGH|1|A (2YGH)	2.6 Å	94	(11) 2YGH|1|A, 3GX5|1|A, 3GX3|1|A, 3IQR|1|A, 3IQP|1|A, 3GX7|1|A, 3IQN|1|A, 2GIS|1|A, 2YDH|1|A, 3GX2|1|A, 3GX6|1|A
14	NR_3.0_60079.1	5X2G|1|B (5X2G)	2.4 Å	93	(2) 5X2G|1|B, 5X2H|1|B
15	NR_3.0_90160.4	5B2P|1|B (5B2P)	1.7 Å	93	(3) 5B2P|1|B, 5B2O|1|B, 5B2Q|1|B
16	NR_3.0_88154.1	4RUM|1|A (4RUM)	2.6 Å	92	(1) 4RUM|1|A
17	NR_3.0_12260.3	3MXH|1|R (3MXH)	2.3 Å	91	(9) 3MXH|1|R, 3MUM|1|R, 3UCZ|1|R, 4YB1|1|R, 3MUR|1|R, 3UD4|1|R, 3MUT|1|R, 3IRW|1|R, 3UCU|1|R
18	NR_3.0_49553.1	3CUL|1|C (3CUL)	2.8 Å	91	(4) 3CUL|1|C, 3CUN|1|C, 3CUL|1|D, 3CUN|1|D
19	NR_3.0_75326.1	1M5K|1|B (1M5K)	2.4 Å	91	(8) 1M5K|1|B, 1M5K|1|E, 1M5O|1|B, 1M5P|1|B, 1M5O|1|E, 1M5V|1|B, 1M5P|1|E, 1M5V|1|E
20	NR_3.0_91539.1	5T83|1|A (5T83)	2.7 Å	90	(1) 5T83|1|A
21	NR_3.0_78797.2	4LVW|1|A (4LVW)	1.8 Å	89	(8) 4LVW|1|A, 4LVX|1|A, 4LVY|1|A, 4LW0|1|A, 4LVV|1|A, 4LVZ|1|A, 3SD3|1|A, 3SD1|1|A
22	NR_3.0_63789.1	3ADD|1|C (3ADD)	2.4 Å	88	(6) 3ADD|1|C, 3ADD|1|D, 3ADB|1|D,

					3ADB|1|C, 3ADC|1|C, 3ADC|1|D
23	NR_3.0_32185.1	3OXE|1|A (3OXE)	2.9 Å	86	(14) 3OXE|1|A, 3OXB|1|A, 3OXB|1|B, 3OXE|1|B, 3OXD|1|A, 3OXD|1|B, 3OXM|1|A, 3OXM|1|B, 3OWI|1|A, 3OWZ|1|A, 3OWI|1|B, 3OWW|1|A, 3OWW|1|B, 3OWZ|1|B
24	NR_3.0_57598.2	3RG5|1|B (3RG5)	2.0 Å	86	(2) 3RG5|1|B, 3RG5|1|A
25	NR_3.0_59099.1	5AOX|1|C (5AOX)	2.0 Å	86	(2) 5AOX|1|C, 5AOX|1|F
26	NR_3.0_01779.1	4FRG|1|B (4FRG)	3.0 Å	84	(2) 4FRG|1|B, 4FRG|1|X
27	NR_3.0_04754.1	5U3G|1|B (5U3G)	2.3 Å	84	(1) 5U3G|1|B
28	NR_3.0_98075.1	2ZZM|1|B (2ZZM)	2.7 Å	84	(1) 2ZZM|1|B
29	NR_3.0_02656.1	4P5J|1|A (4P5J)	2.0 Å	83	(1) 4P5J|1|A
30	NR_3.0_27557.1	4KZD|1|R (4KZD)	2.2 Å	83	(4) 4KZD|1|R, 6B14|1|R, 6B3K|1|R, 4KZE|1|R
31	NR_3.0_31480.1	4YAZ|1|R (4YAZ)	2.0 Å	83	(4) 4YAZ|1|R, 4YAZ|1|A, 4YB0|1|R, 4YB0|1|A
32	NR_3.0_92081.5	5FQ5|1|A (5FQ5)	2.1 Å	83	(8) 5FQ5|1|A, 5FW2|1|A, 5VW1|1|C, 4UN4|1|A, 4UN5|1|A, 5FW3|1|A, 4UN3|1|A, 5FW1|1|A
33	NR_3.0_11709.1	3ZGZ|1|B (3ZGZ)	2.4 Å	82	(14) 3ZGZ|1|B, 4AQ7|1|B, 5OMW|1|B, 3ZGZ|1|E, 4AS1|1|B, 4ARI|1|B, 4AQ7|1|E, 4CQN|1|B,

					3ZJT|1|B, 5OMW|1|E, 3ZJU|1|B, 4ARC|1|B, 4CQN|1|E, 3ZJV|1|B
34	NR_3.0_09775.1	5AH5|1|D (5AH5)	2.1 Å	81	(2) 5AH5|1|D, 5AH5|1|C
35	NR_3.0_13919.1	3AM1|1|B (3AM1)	2.4 Å	81	(1) 3AM1|1|B
36	NR_3.0_33394.2	5B2T|1|A (5B2T)	2.2 Å	81	(5) 5B2T|1|A, 5B2R|1|A, 5B2S|1|A, 4OO8|1|B, 4OO8|1|E
37	NR_3.0_13221.1	1H3E|1|B (1H3E)	2.9 Å	80	(1) 1H3E|1|B
38	NR_3.0_73731.1	3U4M|1|B (3U4M)	2.0 Å	80	(5) 3U4M|1|B, 4QVI|1|B, 3UMY|1|B, 4QG3|1|B, 3U56|1|B
39	NR_3.0_23989.1	2BTE|1|B (2BTE)	2.9 Å	78	(2) 2BTE|1|B, 2BTE|1|E
40	NR_3.0_42241.1	2GDI|1|X (2GDI)	2.1 Å	78	(9) 2GDI|1|X, 2GDI|1|Y, 4NYA|1|A, 2HOJ|1|A, 2HOM|1|A, 2HOL|1|A, 4NYD|1|A, 4NYA|1|B, 2HOO|1|A
41	NR_3.0_56041.1	3AMT|1|B (3AMT)	2.9 Å	78	(1) 3AMT|1|B
42	NR_3.0_72172.1	5CCB|1|N (5CCB)	2.0 Å	78	(2) 5CCB|1|N, 5CCX|1|N
43	NR_3.0_27756.1	3D2V|1|A (3D2V)	2.0 Å	77	(8) 3D2V|1|A, 3D2X|1|A, 3D2V|1|B, 3D2G|1|A, 3D2X|1|B, 2CKY|1|A, 3D2G|1|B, 2CKY|1|B
44	NR_3.0_65284.1	2ZUE|1|B (2ZUE)	2.0 Å	77	(2) 2ZUE|1|B, 2ZUF|1|B
45	NR_3.0_27306.1	4JF2|1|A (4JF2)	2.3 Å	76	(1) 4JF2|1|A
46	NR_3.0_42401.1	4RDX|1|C (4RDX)	2.6 Å	76	(1) 4RDX|1|C
47	NR_3.0_55964.1	5D5L|1|A (5D5L)	2.5 Å	76	(4) 5D5L|1|A, 5D5L|1|D, 5D5L|1|B, 5D5L|1|C

48	NR_3.0_89906.2	5HC9|1|D (5HC9)	2.9 Å	76	(5) 5HC9|1|D, 4WC2|1|B, 5HC9|1|C, 1VFG|1|D, 1VFG|1|C
49	NR_3.0_03715.1	1QU2|1|T (1QU2)	2.2 Å	75	(3) 1QU2|1|T, 1QU3|1|T, 1FFY|1|T
50	NR_3.0_10787.1	5X6B|1|P (5X6B)	2.6 Å	75	(1) 5X6B|1|P
51	NR_3.0_29085.1	1N78|1|C (1N78)	2.1 Å	75	(14) 1N78|1|C, 1N78|1|D, 2DXI|1|C, 1N77|1|C, 2DXI|1|D, 2CV2|1|C, 1N77|1|D, 2CV2|1|D, 2CV1|1|D, 2CV1|1|C, 2CV0|1|C, 2CV0|1|D, 1G59|1|B, 1G59|1|D
52	NR_3.0_5269.1	2DR2|1|B (2DR2)	3.0 Å	75	(1) 2DR2|1|B
53	NR_3.0_57348.1	1GAX|1|D (1GAX)	2.9 Å	75	(4) 1GAX|1|D, 1GAX|1|C, 1IVS|1|C, 1IVS|1|D
54	NR_3.0_58761.1	2CSX|1|C (2CSX)	2.7 Å	75	(4) 2CSX|1|C, 2CT8|1|D, 2CT8|1|C, 2CSX|1|D
55	NR_3.0_73165.2	4PRF|1|B (4PRF)	2.4 Å	75	(14) 4PRF|1|B, 2OJ3|1|B, 1SJF|1|B, 1VC0|1|B, 1VC6|1|B, 1VBZ|1|B, 1SJ4|1|R, 1SJ3|1|R, 2OIH|1|B, 1VBY|1|B, 1VBX|1|B, 1CX0|1|B, 1DRZ|1|B, 1VC7|1|B
56	NR_3.0_04935.1	3AKZ|1|E (3AKZ)	2.9 Å	74	(6) 3AKZ|1|E, 3AKZ|1|F, 3AKZ|1|H, 3AKZ|1|G,

					4YVJ|1|C, 4YVK|1|C
57	NR_3.0_09431.1	4PR6|1|B (4PR6)	2.3 Å	74	(1) 4PR6|1|B
58	NR_3.0_19951.22	4YCO|1|D (4YCO)	2.1 Å	74	(108) 4YCO|1|D, 4YCO|1|F, 4YCO|1|E, 3FOZ|1|C, 4WOI|1|DW, 5NDK|1|X1, 4V9H|1|AV, 3L0U|1|A, 2ZM5|1|C, 5NDK|1|X4, 4V9D|1|BV, 2ZXU|1|C, 3FOZ|1|D, 4WQU|1|BW, 4W2E|1|w, 4WQF|1|BW, 5J4B|1|1y, 6CFJ|1|1w, 5W4K|1|1y, 5WIT|1|1w, 6CFJ|1|1y, 6CFJ|1|2w, 5J4C|1|1w, 5WIS|1|1y, 5J4C|1|1y, 4WQU|1|DW, 5NDK|1|W1, 6CFJ|1|2y, 5WIS|1|1w, 4V51|1|AW, 5J4B|1|1w, 4WQF|1|DW, 5J4C|1|2w, 5J4B|1|2w, 5NDK|1|W4, 5J4B|1|2y, 4WOI|1|AX, 5W4K|1|1w, 5J4C|1|2y, 4W2G|1|AW, 5WIS|1|2y, 5WIT|1|2w, 4V9D|1|AV, 5DOY|1|1y, 2ZM5|1|D, 5W4K|1|2y, 4Y4P|1|1y, 4WSD|1|1K,

4Z3S|1|1y,<br>5DOY|1|1w,<br>4V51|1|CW,<br>5DOY|1|2y,<br>4WSD|1|3L,<br>4W2I|1|AW,<br>4Z3S|1|1w,<br>4Z3S|1|2y,<br>4W2I|1|CW,<br>5W4K|1|2w,<br>1VY4|1|AW,<br>4WPO|1|BY,<br>4Y4P|1|2y,<br>4WSD|1|3K,<br>4W2G|1|CW,<br>4W2I|1|AY,<br>4W2F|1|AW,<br>4Y4P|1|1w,<br>4Z3S|1|2w,<br>4W2I|1|CY,<br>1VY4|1|AY,<br>4W2G|1|AY,<br>4WPO|1|DY,<br>5WIS|1|2w,<br>2ZXU|1|D,<br>5NDK|1|V4,<br>4W2G|1|CY,<br>5WIT|1|1y,<br>4W2F|1|AY,<br>4Y4P|1|2w,<br>5DOY|1|2w,<br>1VY5|1|AW,<br>1VY4|1|CY,<br>4WSD|1|1L,<br>5WIT|1|2y,<br>5NDK|1|V1,<br>4W2F|1|CW,<br>4W2F|1|CY,<br>4WPO|1|BW,<br>1VY7|1|AY,<br>1VY5|1|AY,<br>1VY4|1|CW,<br>1VY7|1|CY,<br>1VY5|1|CW,<br>1VY5|1|CY,<br>4WPO|1|DW,<br>4W2H|1|AY,<br>4WQU|1|BY,<br>4W2H|1|CY,<br>4W2E|1|x,<br>4WQU|1|DY,

					4WQF|1|BY, 4WQF|1|DY, 6AZ1|1|4, 6AZ1|1|2, 3R8O|1|V, 3R8N|1|V, 5AFI|1|y, 5IQR|1|4, 5IQR|1|6
59	NR_3.0_31819.2	1QTQ|1|B (1QTQ)	2.3 Å	74	(20) 1QTQ|1|B, 4JXX|1|B, 1ZJW|1|B, 4V7L|1|AW, 4V7L|1|CW, 1EUY|1|B, 4V7L|1|AY, 4V7L|1|CY, 1QRS|1|B, 1QRT|1|B, 1QRU|1|B, 1GTR|1|B, 2RD2|1|B, 1GTS|1|B, 5NWY|1|M, 2RE8|1|B, 1GSG|1|T, 1O0C|1|B, 1O0B|1|B, 1EXD|1|B
60	NR_3.0_39569.1	4YYE|1|C (4YYE)	2.3 Å	74	(2) 4YYE|1|C, 4YYE|1|D
61	NR_3.0_65073.1	3Q3Z|1|V (3Q3Z)	2.5 Å	74	(2) 3Q3Z|1|V, 3Q3Z|1|A
62	NR_3.0_70420.1	1J1U|1|B (1J1U)	2.0 Å	74	(1) 1J1U|1|B
63	NR_3.0_83386.1	1U0B|1|A (1U0B)	2.3 Å	74	(1) 1U0B|1|A
64	NR_3.0_98593.1	5D8H|1|A (5D8H)	2.8 Å	74	(1) 5D8H|1|A
65	NR_3.0_06496.1	5E6M|1|C (5E6M)	2.9 Å	73	(2) 5E6M|1|C, 5E6M|1|E
66	NR_3.0_45659.1	4ZNP|1|A (4ZNP)	2.9 Å	73	(2) 4ZNP|1|A, 4ZNP|1|B
67	NR_3.0_96162.1	5CZZ|1|B (5CZZ)	2.6 Å	73	(2) 5CZZ|1|B, 5AXW|1|B
68	NR_3.0_30153.3	5AXM|1|P (5AXM)	2.2 Å	72	(10) 5AXM|1|P, 5AXN|1|P, 4TNA|1|A, 1EHZ|1|A, 1I9V|1|A, 1TN1|1|A, 1TN2|1|A, 4TRA|1|A,

					6TNA|1|A, 1TRA|1|A
69	NR_3.0_65123.1	4ZT0|1|D (4ZT0)	2.9 Å	72	(2) 4ZT0|1|D, 4ZT0|1|B
70	NR_3.0_98033.1	2AZX|1|D (2AZX)	2.8 Å	72	(2) 2AZX|1|D, 2AZX|1|C
71	NR_3.0_01439.1	5TPY|1|A (5TPY)	2.8 Å	71	(1) 5TPY|1|A
72	NR_3.0_28348.1	4JXZ|1|B (4JXZ)	2.4 Å	71	(2) 4JXZ|1|B, 4JYZ|1|B
73	NR_3.0_33389.1	2DU3|1|D (2DU3)	2.6 Å	71	(2) 2DU3|1|D, 2DU4|1|C
74	NR_3.0_35542.43	5L4O|1|A (5L4O)	2.8 Å	71	(83) 5L4O|1|A, 5HCQ|1|1x, 5IBB|1|2K, 5HCQ|1|2x, 5IB7|1|2K, 4V8D|1|CC, 5HCR|1|1x, 4Z8C|1|1x, 5J4B|1|1x, 4WSD|1|2L, 4V8D|1|AC, 5IBB|1|2L, 5J4B|1|2x, 4Z8C|1|2x, 4WSD|1|2K, 5J4C|1|1x, 4WPO|1|BX, 5W4K|1|1x, 1VY4|1|AX, 4W2H|1|AX, 5HCR|1|2x, 4LNT|1|XV, 5HD1|1|1x, 5HCP|1|1x, 5HAU|1|1w, 5J4C|1|2x, 4W2I|1|AX, 5WIS|1|1x, 5IB7|1|2L, 4W2G|1|CX, 4LNT|1|QV, 4W2H|1|CX, 5W4K|1|2x, 5HAU|1|2w, 4Y4P|1|1x, 4W2G|1|AX, 5HCP|1|2x, 5DOY|1|1x, 5WIT|1|1x, 5HD1|1|2x,

					4W2I|1|CX, 1VY7|1|AX, 4Z3S|1|1x, 1VY4|1|CX, 4W2F|1|AX, 1VY5|1|AX, 4V51|1|CV, 4WPO|1|DX, 4V9R|1|AX, 4V9R|1|CX, 4W2F|1|CX, 5WIS|1|2x, 4V51|1|AV, 4Y4P|1|2x, 1VY6|1|AX, 4Z3S|1|2x, 1VY6|1|CX, 4WQY|1|BX, 5DOY|1|2x, 5WIT|1|2x, 5F8K|1|1x, 1VY5|1|CX, 4V8B|1|AC, 4V67|1|AY, 4V7L|1|CX, 4V8B|1|CC, 4V7L|1|AX, 5F8K|1|2x, 4V67|1|CY, 4WQY|1|DX, 1VY7|1|CX, 4V8B|1|AD, 4V8B|1|CD, 4V67|1|AZ, 4V67|1|CZ, 6AZ1|1|3, 5AFI|1|v, 5AFI|1|w, 5MDV|1|5, 2FMT|1|D, 2FMT|1|C, 5IQR|1|5, 4BTC|1|V
75	NR_3.0_36247.1	2OIU|1|Q (2OIU)	2.6 Å	71	(1) 2OIU|1|Q
76	NR_3.0_59689.4	4XNR|1|X (4XNR)	2.2 Å	71	(5) 4XNR|1|X, 4TZY|1|X, 4TZX|1|X, 5UZA|1|X, 5SWE|1|X
77	NR_3.0_60142.1	2OIU|1|P (2OIU)	2.6 Å	71	(1) 2OIU|1|P

78	NR_3.0_63265.1	3LA5|1|A (3LA5)	1.7 Å	71	(1) 3LA5|1|A
79	NR_3.0_69516.1	1Y26|1|X (1Y26)	2.1 Å	71	(1) 1Y26|1|X
80	NR_3.0_73445.1	4LX6|1|A (4LX6)	2.2 Å	71	(2) 4LX6|1|A, 4LX5|1|A
81	NR_3.0_74174.1	5KPY|1|A (5KPY)	2.0 Å	71	(1) 5KPY|1|A
82	NR_3.0_79254.1	3KFU|1|L (3KFU)	3.0 Å	71	(4) 3KFU|1|L, 3KFU|1|K, 3KFU|1|N, 3KFU|1|M
83	NR_3.0_90969.1	2ZZN|1|D (2ZZN)	3.0 Å	71	(2) 2ZZN|1|D, 2ZZN|1|C
84	NR_3.0_26998.1	1KXK|1|A (1KXK)	3.0 Å	70	(1) 1KXK|1|A
85	NR_3.0_42502.1	5HR7|1|D (5HR7)	2.4 Å	70	(2) 5HR7|1|D, 5HR7|1|C
86	NR_3.0_93427.1	5UD5|1|C (5UD5)	2.4 Å	70	(4) 5UD5|1|C, 5UD5|1|D, 5V6X|1|C, 5V6X|1|D
87	NR_3.0_45040.1	3EPH|1|E (3EPH)	3.0 Å	69	(2) 3EPH|1|E, 3EPH|1|F
88	NR_3.0_66984.1	3IVN|1|A (3IVN)	2.8 Å	69	(2) 3IVN|1|A, 3IVN|1|B
89	NR_3.0_85174.1	1QF6|1|B (1QF6)	2.9 Å	69	(1) 1QF6|1|B
90	NR_3.0_88566.1	3MOJ|1|A (3MOJ)	2.9 Å	69	(1) 3MOJ|1|A
91	NR_3.0_92167.1	5OB3|1|A (5OB3)	2.0 Å	69	(1) 5OB3|1|A
92	NR_3.0_03710.1	1C0A|1|B (1C0A)	2.4 Å	68	(3) 1C0A|1|B, 1EFW|1|D, 1EFW|1|C
93	NR_3.0_19188.1	4QEI|1|C (4QEI)	2.9 Å	68	(1) 4QEI|1|C
94	NR_3.0_40252.1	2QUS|1|A (2QUS)	2.4 Å	68	(2) 2QUS|1|A, 2QUS|1|B
95	NR_3.0_56345.1	5SWD|1|B (5SWD)	2.5 Å	68	(4) 5SWD|1|B, 5E54|1|B, 5E54|1|A, 5SWD|1|A
96	NR_3.0_65615.1	5HR6|1|C (5HR6)	2.9 Å	68	(2) 5HR6|1|C, 5HR6|1|D
97	NR_3.0_70496.1	1Y27|1|X (1Y27)	2.4 Å	68	(1) 1Y27|1|X
98	NR_3.0_72021.1	4PQV|1|A (4PQV)	2.5 Å	68	(1) 4PQV|1|A
99	NR_3.0_24819.2	1IL2|1|C (1IL2)	2.6 Å	67	(9) 1IL2|1|C, 1IL2|1|D, 2TRA|1|A, 3TRA|1|A, 1ASZ|1|S, 1ASY|1|S, 1ASZ|1|R, 1ASY|1|R, 1VTQ|1|A
100	NR_3.0_27778.1	5WT1|1|C (5WT1)	2.6 Å	67	(2) 5WT1|1|C,

					5WT1|1|F
101	NR_3.0_76114.1	3RKF|1|C (3RKF)	2.5 Å	67	(4) 3RKF|1|C, 3RKF|1|B, 3RKF|1|A, 3RKF|1|D
102	NR_3.0_89429.3	4FEN|1|B (4FEN)	1.4 Å	67	(27) 4FEN|1|B, 4FEO|1|B, 4FEL|1|B, 4FEP|1|B, 4FEJ|1|B, 4FE5|1|B, 2XNZ|1|A, 2XNW|1|A, 3GOT|1|A, 2G9C|1|A, 3GAO|1|A, 3GER|1|A, 2EES|1|A, 3GOG|1|A, 3DS7|1|A, 2EET|1|A, 3FO4|1|A, 2EEV|1|A, 3DS7|1|B, 3FO6|1|A, 3G4M|1|A, 3GES|1|A, 2B57|1|A, 2EEW|1|A, 2EEU|1|A, 2XO1|1|A, 1U8D|1|A
103	NR_3.0_21705.1	3SKI|1|A (3SKI)	2.3 Å	66	(8) 3SKI|1|A, 3SKI|1|B, 3SLM|1|B, 3SKZ|1|A, 3SLM|1|A, 3SLQ|1|A, 3SKZ|1|B, 3SLQ|1|B
104	NR_3.0_34221.1	1B23|1|R (1B23)	2.6 Å	66	(1) 1B23|1|R
105	NR_3.0_26284.1	1H4S|1|T (1H4S)	2.9 Å	65	(2) 1H4S|1|T, 1H4Q|1|T
106	NR_3.0_30439.1	3EGZ|1|B (3EGZ)	2.2 Å	65	(1) 3EGZ|1|B
107	NR_3.0_60469.1	4N0T|1|B (4N0T)	1.7 Å	65	(3) 4N0T|1|B, 5TF6|1|B, 5TF6|1|D
108	NR_3.0_47330.1	3SKL|1|B (3SKL)	2.9 Å	64	(4) 3SKL|1|B, 3SKL|1|A, 3SKW|1|A,

					3SKW|1|B
109	NR_3.0_48352.1	4XWF|1|A (4XWF)	1.8 Å	64	(2) 4XWF|1|A, 4XW7|1|A
110	NR_3.0_55424.1	3NKB|1|B (3NKB)	1.9 Å	64	(1) 3NKB|1|B
111	NR_3.0_63919.1	5B63|1|D (5B63)	3.0 Å	64	(2) 5B63|1|D, 5B63|1|B
112	NR_3.0_83181.1	2DLC|1|Y (2DLC)	2.4 Å	64	(1) 2DLC|1|Y
113	NR_3.0_99632.2	1YFG|1|A (1YFG)	3.0 Å	64	(1) 1YFG|1|A
114	NR_3.0_82534.1	1F7U|1|B (1F7U)	2.2 Å	63	(2) 1F7U|1|B, 1F7V|1|B
115	NR_3.0_03470.1	1SER|1|T (1SER)	2.9 Å	62	(1) 1SER|1|T
116	NR_3.0_06843.2	1EVV|1|A (1EVV)	2.0 Å	62	(4) 1EVV|1|A, 1TTT|1|F, 1TTT|1|D, 1TTT|1|E
117	NR_3.0_51962.2	4YCP|1|B (4YCP)	2.6 Å	62	(1) 4YCP|1|B
118	NR_3.0_76901.1	5BTP|1|B (5BTP)	2.8 Å	62	(2) 5BTP|1|B, 5BTP|1|A
119	NR_3.0_39792.1	4U7U|1|L (4U7U)	3.0 Å	61	(2) 4U7U|1|L, 4U7U|1|X
120	NR_3.0_46720.2	5DDP|1|A (5DDP)	2.3 Å	61	(4) 5DDP|1|A, 5DDP|1|B, 5DDR|1|B, 5DDR|1|A
121	NR_3.0_99751.1	2HVY|1|E (2HVY)	2.3 Å	61	(1) 2HVY|1|E
122	NR_3.0_23049.1	1KUQ|1|B (1KUQ)	2.8 Å	60	(3) 1KUQ|1|B, 1F7Y|1|B, 1DK1|1|B
123	NR_3.0_34126.1	3HAX|1|E (3HAX)	2.1 Å	60	(1) 3HAX|1|E
124	NR_3.0_70313.2	5H9F|1|L (5H9F)	2.5 Å	60	(1) 5H9F|1|L
125	NR_3.0_74932.1	6B44|1|M (6B44)	2.9 Å	60	(1) 6B44|1|M
126	NR_3.0_76909.1	3RW6|1|H (3RW6)	2.3 Å	60	(2) 3RW6|1|H, 3RW6|1|F
127	NR_3.0_33278.1	5LYS|1|B (5LYS)	2.3 Å	59	(6) 5LYS|1|B, 5LYV|1|B, 5LYS|1|A, 5LYU|1|B, 5LYU|1|A, 5LYV|1|A
128	NR_3.0_48748.1	5T5A|1|A (5T5A)	2.0 Å	59	(1) 5T5A|1|A
129	NR_3.0_52315.1	4M4O|1|B (4M4O)	2.0 Å	59	(1) 4M4O|1|B
130	NR_3.0_39651.1	3HJW|1|D (3HJW)	2.4 Å	58	(6) 3HJW|1|D, 3LWV|1|D, 3LWR|1|D, 3LWP|1|D, 3LWO|1|D, 3LWQ|1|D
131	NR_3.0_74849.1	1MMS|1|C (1MMS)	2.6 Å	58	(2) 1MMS|1|C, 1MMS|1|D

132	NR_3.0_36634.1	2QUW|1|B (2QUW)	2.2 Å	57	(2) 2QUW|1|B, 2QUW|1|D
133	NR_3.0_95614.1	1HC8|1|C (1HC8)	2.8 Å	57	(6) 1HC8|1|C, 1HC8|1|D, 1Y39|1|C, 1Y39|1|D, 1QA6|1|C, 1QA6|1|D
134	NR_3.0_26186.1	4JRC|1|B (4JRC)	2.7 Å	56	(2) 4JRC|1|B, 4JRC|1|A
135	NR_3.0_68503.2	3F4H|1|Y+3F4H|1|X (3F4H)	3.0 Å	56	(2) 3F4H|1|Y+3F4H|1|X, 6BFB|1|Y+6BFB|1|X
136	NR_3.0_26582.1	4K27|1|U (4K27)	2.4 Å	55	(1) 4K27|1|U
137	NR_3.0_46884.1	1MZP|1|B (1MZP)	2.7 Å	55	(1) 1MZP|1|B
138	NR_3.0_55702.1	1C9S|1|W (1C9S)	1.9 Å	55	(1) 1C9S|1|W
139	NR_3.0_18889.3	5C45|1|X+5C45|1|Y (5C45)	2.9 Å	54	(3) 5C45|1|X+5C45|1|Y, 5KX9|1|X+5KX9|1|Y, 2YIE|1|X+2YIE|1|Z
140	NR_3.0_19502.2	4RGE|1|C (4RGE)	2.9 Å	54	(4) 4RGE|1|C, 4RGE|1|B, 4RGE|1|A, 5DUN|1|A
141	NR_3.0_24202.1	4PKD|1|V (4PKD)	2.5 Å	54	(1) 4PKD|1|V
142	NR_3.0_79184.1	4GCW|1|B (4GCW)	3.0 Å	53	(2) 4GCW|1|B, 2FK6|1|R
143	NR_3.0_79951.1	4OJI|1|A (4OJI)	2.3 Å	53	(1) 4OJI|1|A
144	NR_3.0_58167.1	3E5C|1|A (3E5C)	2.3 Å	52	(3) 3E5C|1|A, 3E5F|1|A, 3E5E|1|A
145	NR_3.0_82984.1	4ENC|1|A (4ENC)	2.3 Å	52	(5) 4ENC|1|A, 4ENB|1|A, 3VRS|1|A, 4ENA|1|A, 4EN5|1|A
146	NR_3.0_83080.1	2QWY|1|C (2QWY)	2.8 Å	52	(3) 2QWY|1|C, 2QWY|1|A, 2QWY|1|B
147	NR_3.0_83027.1	3NPQ|1|A (3NPQ)	2.2 Å	51	(4) 3NPQ|1|A, 3NPQ|1|C, 3NPQ|1|B, 3NPN|1|A

The columns are number, equivalence class, representative structure, resolution, nucleotide length, and class member, respectively.
